# Comparing microbial populations from diverse hydrothermal features in Yellowstone National Park: hot springs and mud volcanoes

**DOI:** 10.3389/fmicb.2024.1409664

**Published:** 2024-06-27

**Authors:** Laura Rowe, Scot E. Dowd, Kelly Davidson, Claire Kovarik, Michayla VanAken, Alyssa Jarabek, Churro Taylor

**Affiliations:** ^1^Department of Chemistry, Eastern Kentucky University, Richmond, KY, United States; ^2^Department of Chemistry, Valparaiso University, Valparaiso, IN, United States; ^3^Molecular Research LP (MR DNA Lab), Shallowater, TX, United States

**Keywords:** extremophiles, hot spring, mud volcano, Yellowstone National Park, microbial diversity, 16S rRNA sequencing, pH, temperature

## Abstract

Geothermal features, such as hot springs and mud volcanoes, host diverse microbial life, including many extremophile organisms. The physicochemical parameters of the geothermal feature, such as temperature, pH, and heavy metal concentration, can influence the alpha and beta diversity of microbial life in these environments, as can spatiotemporal differences between sites and sampling. In this study, water and sediment samples were collected and analyzed from eight geothermal sites at Yellowstone National Park, including six hot springs, a mud volcano, and an acidic lake within the same week in July 2019, and these geothermal sites varied greatly in their temperature, pH, and chemical composition. All samples were processed and analyzed with the same methodology and taxonomic profiles and alpha and beta diversity metrics determined with 16S rRNA sequencing. These microbial diversity results were then analyzed with respect to pH, temperature, and chemical composition of the geothermal features. Results indicated that predominant microbial species varied greatly depending on the physicochemical composition of the geothermal site, with decreases in pH and increases in dissolved heavy metals in the water corresponding to decreases in alpha diversity, especially in the sediment samples. Similarly, sites with acidic pH values had more similar microbial populations (beta diversity) to one another than to relatively neutral or alkaline pH geothermal sites. This study suggests that pH and/or heavy metal concentration is a more important driver for microbial diversity and population profile than the temperature for these sites and is also the first reported microbial diversity study for multiple geothermal sites in Yellowstone National Park, including the relatively new mud volcano Black Dragon’s Caldron, which erupted in 1948.

## 1 Introduction

Extreme environments, such as those found in terrestrial hot springs, mud pots, geysers, and steam vents (fumaroles), host a wide variety of unique extremophile organisms that have evolved to survive the extremes in temperature, pH, and chemical composition often found in these geothermal features. Yellowstone National Park (YNP) is centered over the Yellowstone Caldera, the largest super-volcano in North America. Due to the location of YNP over the active volcanism of the Yellowstone hotspots, YNP is home to over half of world’s known geothermal features (Fournier, 1989). The physical and chemical properties of the geothermal features of YNP vary widely both by region within YNP and between individual features within the same region. Due to the variety of conditions at YNP, the geothermal sites have been analyzed in terms of not only their extremophile microbial diversity but also as a model for prebiotic chemistry in the Archaean age and as a terrestrial analog site for early Mars in astrobiology research (Bowen De León et al., 2013; Inskeep et al., 2015; Jiang, 2015; Colman et al., 2016; Jiang and Takacs-Vesbach, 2017; Tank et al., 2017; Gonsior et al., 2018; Stone et al., 2018; Aerts et al., 2019; Bennett et al., 2020; Colman et al., 2021; Bennett et al., 2022; Fernandes-Martins et al., 2023).

**Figure 1:**
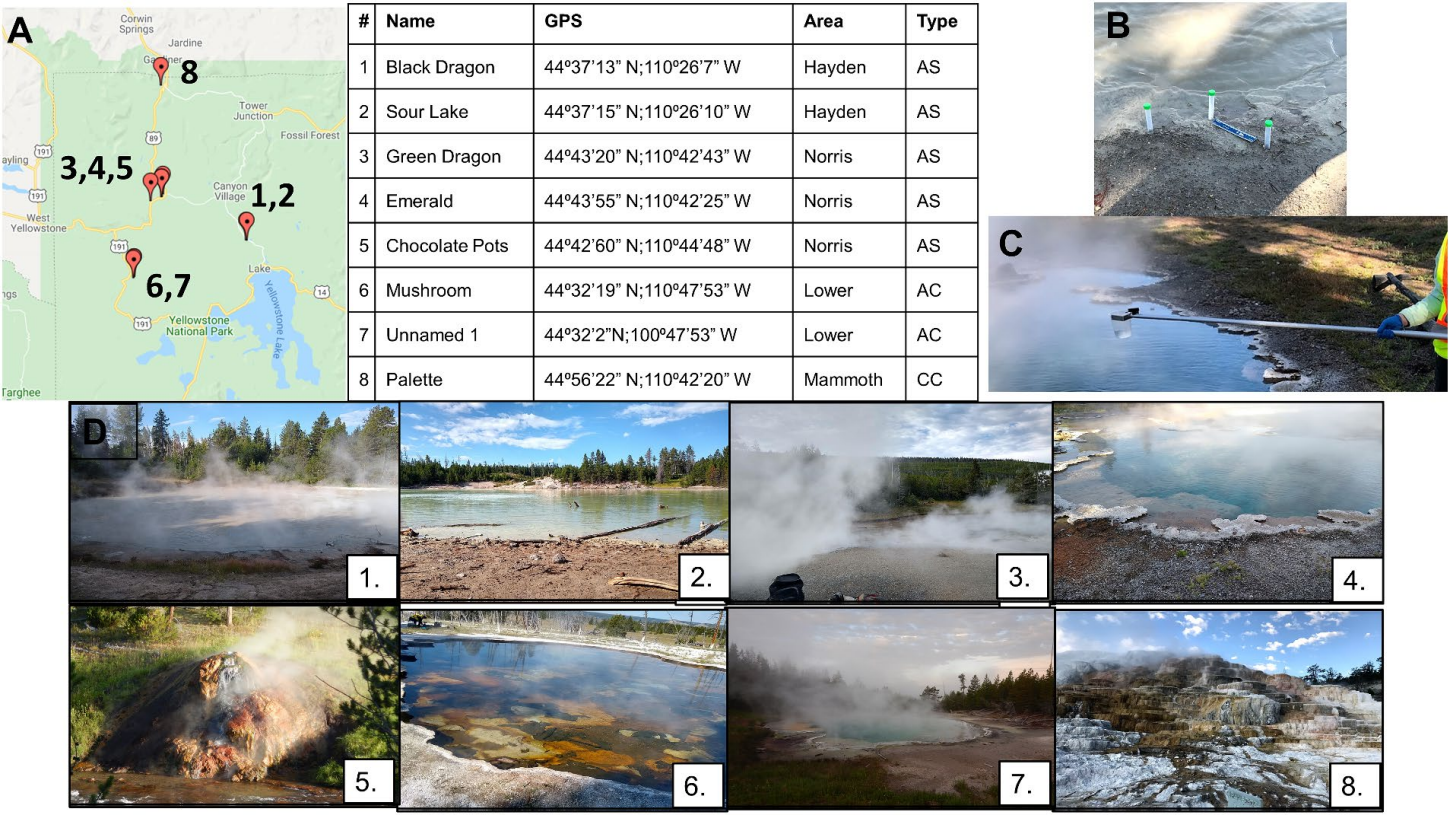
Locations of all geothermal sites sampled **(A)** with GPS coordinates. Panel **(A)** also shows which basin the sampling was in (Hayden, Norris, Lower, Mammoth Basins) and the type of hot spring/geothermal site, with AS meaning acid sulfate, AC being alkaline chloride, and CC calcium containing. Soil samples were collected at the interface between the water and soil **(B)**, and water samples were collected between 1 and 3 feet from the surface **(C)**. Photographs of all sample sites **(D)**: Black Dragon’s Caldron (1), Sour Lake (2), Green Dragon Springs (3), Emerald Springs (4), Chocolate Pots Springs (5), Mushroom Springs (6), Unnamed Hot Springs (7), and Palette Springs (8).

The rhyolite- and basalt-hosted hydrothermal systems in YNP are usually characterized by two end members: (1) acid-sulfate boiling pools, mud pots, and fumaroles and (2) alkaline-chloride silica-depositing hot springs (Fournier, 1989; Des Marais and Walter, 2019). These two end members mix to generate acid-sulfate-chloride springs. The hydrothermal areas of YNP are generally separated into eight different geyser basin regions that encompass different geochemical features: Mammoth Hot Springs, Norris Geyser Basin, Artist’s Paint Pots, Lower Geyser Basin, Midway Geyser Basin, Upper Geyser Basin, West Thumb Geyser Basin, and Hayden Valley Geyser Basin (Figure 1). In Mammoth Hot Springs, calcium carbonate, and travertine hot springs predominate, acid sulfate geothermal features predominate in the Norris and Hayden Valley Geyser Basins, whereas the other regions contain a mixture of both acid sulfate and alkaline chloride geothermal features. The very different types of geothermal features of YNP have evolved diverse microbial populations that are resistant to multiple biocidal conditions. Previous research has shown that the geothermal features of YNP are organic chemodiversity hotspots, and the varying conditions of the different features produce wildly different microbial populations, even when such features are geographically very close to one another (Gonsior et al., 2018).

Many studies have assessed the microbial diversity of different YNP geothermal features, often using next-generation sequencing methods and 16S rRNA analysis (Barns et al., 1994; Reysenbach et al., 1994, 2000; Blank et al., 2002; Meyer-Dombard et al., 2005; Parenteau and Cady, 2010; Bowen De León et al., 2013; Sharp et al., 2014; Colman et al., 2016, 2021, 2022; Dong et al., 2019; Hamilton et al., 2019; Bennett et al., 2020, 2022; Massello et al., 2020; Podar et al., 2020; Ando et al., 2021; Gonzalez-Nayeck et al., 2022; Peach et al., 2022; Fernandes-Martins et al., 2023). These studies highlight the enormous variation of microbial life depending not only between the individual geothermal site studied but also on the area of the individual geothermal site sampled (deep vs. shallow, close to source vs. downstream from source, etc.) and the season and year the site was sampled. Moreover, different groups often choose different DNA extraction methods and different 16S rRNA sequencing pipelines, and these different experimental parameters have been shown to affect the microbial population distribution identified from environmental samples (Lauber et al., 2010, 2013; Hinlo et al., 2017; Colman et al., 2021). The uniqueness of this study is that eight different geothermal sites at YNP with wide-ranging pH, temperature, and chemical compositions were all sampled within the same week, and all samples underwent identical storage, processing, DNA extraction, and 16S rRNA sequencing protocols. Moreover, every effort was made to collect all samples from the same general area of the geothermal site; 1–1.5 m from the shore and 0.3–0.6 m depth for water samples and areas close to the shore that would intermittently get wet from the geothermal source depending on rainfall for the sediment samples. This temporal, sampling, and processing homogeneity reduces variables that can affect taxonomic distribution and allows a more robust analysis of the effect pH, temperature, and chemical composition have on microbial diversity in extreme environments found in these sites.

## 2 Materials and methods

### 2.1 Description of sampling sites and sampling

All samples at YNP were collected between the dates of 23–30 July 2019. Water samples were collected with sterile 600-mL ladles hung from a Conbar metal telescopic pole fitted with a swivel adapter (Forestry Supply, Reedsburg, WI). Water collection occurred from approximately 0.3 to 0.6 m deep to the surface a minimum of 1 m from the shore, when possible. Water samples were stored in sterile Nunc polypropylene centrifuge tubes of varying sizes, and filled approximately three-fourths full to ensure freezing expansion would not break the storage containers (Thermo Fisher Scientific, Waltham, MA). Soil samples were collected at the interface where the water met the soil, with three different collection sites approximately 0.3 m away from one another at each location (Figure 1). Soil samples were collected using autoclaved scoopulas and stored in sterile 14-mL Nunc polypropylene centrifuge tubes (Thermo Fisher Scientific, Waltham, MA). Water and soil samples for microbial diversity analysis were immediately stored in a cooler with ice packs (Arctic Ice Tundra Series ice packs, −15°C) following collection and remained on ice packs no more than 1 h before they were transferred to a cooler containing dry ice. Samples remained frozen on dry ice or in a −80°C ultracold freezer until just before sample preparation for analysis.

The GPS coordinates and location of the sampling sites in YNP are shown in Figure 1, and photographs of the individual sampling sites are shown in Figure 1. Samples were taken from travertine pools of Palette Springs in the Mammoth Hot Springs area (30 July 2019, early morning), a calcium carbonate spring. Due to the shallowness of these pools (< 0.15 m depth) and the need to not excessively disturb the sediment, the water samples were collected from 2 to 5 cm deep to the surface for this sampling site. Three different hot spring sites were sampled in the Norris Geyser Basin: Green Dragon hot spring (23 July 2019, early morning) and Emerald hot springs (24 July 2019, early morning) both acidic hot springs with temperatures greater than 60°C, and Chocolate Pots hot spring (25 July 2019, early morning), which was not as hot or acidic as Green dragon or Emerald hot springs. Chocolate Pots also had only very shallow pools of water, such that the water samples were collected no more than 2.5 cm below the surface. Mushroom hot springs (26 July 2019, afternoon) and an Unnamed one hot spring close to Octopus hot spring (26 July 2019, early morning) in the Lower Geyser Basin were sampled, with these sites having alkaline pH and hot temperatures. The Black Dragon’s Caldron mud pot (30 July 2019, afternoon) and neighboring Sour Lake (20 July 2019, afternoon) in Hayden Valley Basin were sampled, with both having very acidic pH values and moderate temperatures. All sites besides Black Dragon’s Caldron and Sour Lake are considered hot springs, whereas Black Dragon’s Caldron and Sour Lake are in the Mud Volcano Group of Hayden Valley, with Black Dragon being a bubbling mud pot.

### 2.2 Physicochemical analysis

The temperature, pH, dissolved oxygen (O_2_), dissolved sulfide gas (H_2_S), and dissolved chlorine (Cl_2_) levels of the water in the hot springs or mud pots were determined on-site immediately prior to sample collection. The surface temperature of each geothermal feature was initially estimated with a high-temperature infrared thermometer (EXTECH, Pittsburgh, PA), and the final temperature of water was determined with the immersion of a Traceable digital pocket thermometer with a 3-m probe (Traceable, Webster, TX). Temperature measurements were taken 3 times in a 1 m area where water sampling was to occur, and the average and standard deviation of the immersed temperature were reported. The pH of the water at each sampling site was also completed two to three times, with three different water samples at the area to be sampled, using the Hach Pocket Pro pH meter (Hach, Loveland, CO), with the mean pH value reported. A Hach handheld colorimeter DR900 was used for dissolved oxygen, sulfide gas, and chlorine levels determinations. Each test was completed either two or three times, with freshly collected samples utilized for each replicate. The vacuum ampules for the DPP total chlorine and high-range dissolved oxygen Hach tests, along with vacuum ampule blanks (Hach, Loveland, CO), were used for dissolved chlorine and oxygen levels in the water, respectively, using the manufacturer protocols (Montgomery et al., 1964; Wilde, 1991). The sulfide methylene blue method using Sulfide 1 and Sulfide 2 reagents and the Hach colorimeter were used for H_2_S concentration determination (Hach, Loveland, CO) using the manufacturer’s protocol (Lindsay and Baedecker, 1988). Samples from the Black Dragon’s Caldron did not have their dissolved oxygen, chloride, or sulfide gas concentrations determined on-site due to the excessive amount of unsettled sediment in the sample not permitting colorimetric analysis with the Hach colorimeter.

The ion and metal concentrations of the water samples were determined at the Water Quality Core Facility at the University of Tennessee, Knoxville. Dissolved metals in water samples were measured by inductively coupled argon plasma optical emission spectrometry (ICP-OES), Thermo Scientific iCAP 7,400 ICP spectrometer, and composition of anions and cation in the water samples was determined with ion chromatography (IC) using a Thermo Scientific/Dionex ICS-2100 (anions) and ICS-1100 (cations) with background suppression. Samples for both ICP-OES and IC were stored in sterile 50-mL Nunc polypropylene centrifuge tubes (Thermo Fisher Scientific, Waltham, MA). For ICP-OES analysis, water samples were thawed, filtered through sterile 0.45-μm syringe filters, and acidified to a pH < 2 using dropwise addition of ultra-high-purity ultratrace metal grade nitric acid (Thermo Fisher Scientific, Waltham, MA). Samples were kept cold until ICP-OES analysis (<1 week). For a total of eight quality control samples, three calibration standards, two calibration check standards, and three calibration blank checks were included in the ICP analysis. Samples were run in ICP undiluted and at 1:100 dilution. Concentrations of sodium, potassium, magnesium, calcium, aluminum, copper, iron, manganese, silicon, zinc, cadmium, cobalt, chromium, nickel, and lead (Na, K, Mg, Ca, Al, Cu, Fe, Mn, Si, Zn, Cd, Co, Cr, Ni, and Pb, respectively) in the water samples were determined with ICP-OES.

For IC, water samples were thawed and filtered through sterile 0.2-μm nitrocellulose syringe filters and kept cold (4°C) until analysis (<1 week) (Corning, Corning, NY). For the IC analysis, for a total of 16 quality control samples, three calibration standards were used for each IC mode (three for anions and three for cations), two calibration check standards for each mode, and three calibration blank checks were used for each mode. Concentrations of ammonium cation and fluoride, hydrogen phosphate, nitrite, nitrate, sulfate, and chloride anions (NH_4_^+^, F-, HPO_4_^−^, NO_2_^−^, NO_3_^−^, SO_4_^2−^, Cl^−^, respectively) of the water samples were determined with IC.

### 2.3 DNA extraction, 16S rRNA sequencing, and analysis

DNA was extracted from the soil samples using DNeasy PowerSoil kits (Qiagen, Germantown, MD), with soil samples being thawed just before analysis. Soil samples from all three sampling locations at each site were thawed, and 0.5 g of soil from each sampling site was added, with an autoclaved scoopula, to a cleaned mortar and pestle and mixed and ground with the scoopula and pestle. The mortar and pestle were cleaned with soap and water, ethanol, and sterile water and allowed to dry prior to use with soil from each site. After the soil from the three sampling locations was thoroughly mixed, 0.25 g of the soil sample was added to the PowerBead tube from the kit. Kit procedures were followed per the manufacturer’s protocol, with 100 μL of Solution C6 used for elution. The concentration of DNA was determined using an Invitrogen Nanodrop One Spectrophotometer (Thermo Scientific, Waltham, MA). The extracted DNA was stored at −80°C until 16S rRNA sequencing. DNA extraction and concentration from both the soil and water samples were completed within 6 months of collection (July 2019) and stored at −80°C until 16S rRNA sequencing. However, due to COVID-19 lockdown restrictions (beginning in March 2020), the 16S rRNA sequencing was not completed until October 2021.

DNA extraction from water samples was completed using the DNeasy Blood and Tissue Kit (Qiagen, Germantown, MD) and manufacturer’s protocols, with previously established additional procedures for the water samples, as follows: Just prior to analysis, the water sample was thawed at room temperature and a volume of 50 mL of the water from the site was filtered through a 47 mM, 0.45-μm pore size sterile Whatman cellulose nitrate filter (GE Healthcare, Chicago, IL) using a vacuum suction filtration apparatus. The glassware of the filtration apparatus was soaked in a 10% bleach solution for 10 min, then soaked in a sterile EZ Pure water solution for 10 min, and thoroughly rinsed with sterile EZ Pure water prior to use. After 50 mL of water was filtered, the filter was folded with sterile tweezers and placed into a sterile 2-mL microcentrifuge tube. If the filter was not immediately processed, and then, it was stored at −20°C for no more than 1 week; 567 μL of ATL buffer from the kit and 63 μL of proteinase K were added to the microcentrifuge tube containing the filter and incubated in a water bath at 65°C for 1 h; 630 μL of AL buffer was then added to the tube with the filter and vortexed for 5 s. Approximately one-third of the mixture from this filter containing microcentrifuge tube was then pipetted onto the top of a spin column from the kit, centrifuged at 6,000 g for 1 min, and the flow through discarded. This was repeated until all the liquid from the filter-containing tube had been spun through the same spin column. The filter was not placed in a spin column. The spin column was placed in a fresh 2-mL collection tube, 500 μL of buffer AW1 added to the top of the spin column and centrifuged for 1 min at 6,000 g. Flow through was discarded; 500 μL of Buffer AW2 was added and centrifuged for 3 min at 16,000 g, flow through was discarded. The spin column was transferred to a new sterile 2-mL microcentrifuge tube, and DNA was eluted with 200 μL of Buffer AE, incubating for 1 min at room temperature, and centrifuging for 1 min at 6,000 g. Flow through was kept, and this step was repeated for a total of 400 μL of collected DNA solution (Hinlo et al., 2017). In most cases, the DNA concentration was not high enough for 16S rRNA sequencing (>5 ng DNA per μL) and DNA in the solution was then concentrated using the manufacturer’s protocols in an Abcam DNA concentration Kit (Abcam, Waltham, MA). The concentration of DNA was determined using an Invitrogen Nanodrop One Spectrophotometer (Thermo Scientific, Waltham, MA). The extracted DNA was stored at −80°C until 16S rRNA sequencing.

Extracted DNA samples from the soil and water samples were stored in sterile 2.0-mL microcentrifuge tubes and shipped on ice to Molecular Research LP (MR DNA™ www.mrdnalab.com) for 16S rRNA sequencing. The 16S rRNA V4 variable region was amplified using 515F 5’GTGYCAGCMGCCGCGGTAA3’ and 806RB 5’GGACTACNVGGGTWTCTAAT3’ v4 16s primer set in a single-step 30-cycle PCR with HotStarTaq Plus Master Mix Kit (Qiagen, USA): 94°C, 3 min; 30 cycles of 94°C, 30 s; 53°C, 40 s; 72°C 1 min; 72°C, 5 min on an Illumina MiSeq (Illumina Inc., San Diego, CA). The PCR products were checked for amplification success with a 2% agarose gel. Samples were then purified with AMPure XP beads (Beckman Coulter, Life Sciences, Indianapolis, IN), and the PCR products were used to prepare an Illumina DNA library for abTEFAP diversity assay with Illumina MiSeq sequencing platform (Dowd et al., 2008; Palavesam et al., 2012; Mukherjee et al., 2014). The sequence data were processed with standard workflows through Qiime2 including demux, dada2, SEPP, Silva, background filtering, and core metrics (Qiime, 2019). Primer-free sequences were imported into Qiime2 and inspected with demux. The sequences were then processed/denoised with DADA2 to remove contamination and PCR chimeras, trim reads, correct errors, and merge read pairs. SEPP was used to align sequences, and Silva was used for taxonomic classification. Qiime2 was used for determining non-phylogenetic and phylogenetic alpha diversity metrics: observed features, Shannon’s index, and Faith’s phylogenetic alpha diversity. Phylogenetic and non-phylogenetic beta diversity metrics, weighted and unweighted UniFrac, Bray–Curtis dissimilarity, and Jaccard similarity beta diversity indexes were also calculated in Qiime2 and displayed with a 3D PCoA Emperor plot. Unprocessed sequences were uploaded into the NCBI SRA database, and project name and accession numbers are given in Supplementary material.

## 3 Results

### 3.1 Temperature, pH, and chemical composition of geothermal sites

In total, eight geothermal sites were selected at YNP for their variety in temperature, pH, and chemical composition (Figure 1; Tables 1, 2; Supplementary Tables 1, 2). The numbering of the sites is from least to greatest pH except Palette Springs (#8) being listed after Unnamed Hot Spring 1, due to it being the only calcium carbonate hot spring. The pH, temperature, dissolved O_2_, Cl_2_, and H_2_S gas concentrations of the water for all sites were determined on-site during sample collection, except Black Dragon’s Caldron dissolved gases were not determined due to the extremely sediment-rich water sample negating colorimetric analysis (Supplementary Table 1). The cation and anion concentrations determined with IC are given in Supplementary Table 2, and the dissolved metals determined with ICP are given in Table 2. In addition to metals listed in Table 2, Cd, Co, Cr, Ni, and Pb concentrations were also determined with ICP-OES, but Cd, Co, and Pb concentrations were found to be zero for all sites. Cr and Ni were found to be at zero concentrations at all sites except Black Dragon’s Caldron and Sour Lake, with Black Dragon’s Caldron having 0.027 mg/L of Cr and 0.006 mg/L of Ni and Sour Lake having 0.019 mg/L of Cr and 0.005 mg/L or Ni.

**Table 1:** Kingdom diversity, expressed as a percentage, in sediment (top) and water (bottom) samples from geothermal sites.

Name	pH	Temperature °C	Bacteria (%)	Archaea (%)	Viridiplantae (%)	Fungi (%)	Eukaryote (%)
Black Dragon’s Caldron	1.93	48.0	19.47	80.46	0.06	0.00	0.00
			87.76	12.17	0.00	0.05	0.01
Sour Lake	2.15	21.7	51.42	0.30	0.85	32.38	15.04
			97.95	0.17	1.15	0.44	0.29
Green Dragon	2.93	93.0	58.81	36.15	1.72	0.00	3.33
			83.73	13.44	1.42	0.63	0.78
Emerald	3.31	77.6	93.04	2.90	3.14	0.17	0.90
			81.36	17.83	0.78	0.00	0.03
Chocolate Pots	6.20	50.8	93.56	4.41	1.10	0.72	0.21
			97.99	1.65	0.07	0.02	0.26
Mushroom	8.09	64.5	99.88	0.06	0.01	0.01	0.03
			99.86	0.14	0.00	0.00	0.00
Unnamed 1	8.59	84.5	98.85	0.85	0.05	0.15	0.10
			99.71	0.17	0.04	0.01	0.06
Palette	8.31	25.8	94.49	0.65	0.21	0.11	4.55
			99.10	0.74	0.02	0.06	0.07

Temperature and pH reported are that of the water at the geothermal site.

**Table 2:** Metal concentrations of geothermal sites as determined by ICP-OES.

Name	Na (mg/L)	K (mg/L)	Mg (mg/L)	Ca (mg/L)	Al (mg/L)	Fe(mg/L)	Mn (mg/L)	Si (mg/L)	Zn (mg/L)
Black Dragon’s Caldron	93.56	29.31	36.43	61.17	32.48	52.220	1.25	0.08	0.16
Sour Lake	111.91	14.38	9.612	32.33	14.49	9.085	0.71	0.02	0.09
Green Dragon	177.37	37.93	0.14	2.31	1.55	0.616	0.08	0.97	0.02
Emerald	245.15	28.83	0.09	1.73	0.99	0.170	0.02	1.14	0.01
Chocolate Pots	68.96	12.22	1.11	11.65	-	0.013	0.76	0.38	-
Mushroom	180.35	10.86	-	0.15	-	-	-	4.37	-
Unnamed 1	218.04	9.48	-	0.21	-	-	-	5.74	-
Palette	92.561	38.82	56.85	136.40	-	-	-	11.54	-

Cd, Co, and Pb concentrations were found to be zero, or below detection limits for all sites. Cr and Ni were found to be at zero concentrations, or below detection limits, at all sites except Black Dragon’s Caldron and Sour Lake, with Black Dragon’s Caldron having 0.027 mg/L of Cr and 0.006 mg/L of Ni, and Sour Lake having 0.019 mg/L of Cr and 0.005 mg/L or Ni.

Black Dragon’s Caldron is a bubbling mud pot in the Hayden Valley Basin, and Sour Lake is a neighboring acidic lake. These two features exhibited the most acidic pH (1.93 and 2.15, respectively), moderate to cool temperatures (48.0°C and 21.7°C), and very high heavy metal concentrations (Fe, Al, Mn, and Zn; Table 2). Green Dragon Springs and Emerald Springs are hot, acidic hot springs in the Norris Geyser Basin. Green Dragon Hot Springs had the highest temperature of any sampled site (93.0°C) and a pH of 2.93, whereas the slightly less hot Emerald Springs had a temperature of 77.6°C and a pH of 3.31 (Table 1). Green Dragon and Emerald Hot Springs both had high sulfate ion, chloride anion, sodium, and potassium concentrations and low dissolved oxygen and chloride levels.

Chocolate Pots hot springs are also in the Norris Geyser Basin but had a more neutral pH (6.20) and milder temperature (50.8°C). Mushroom Hot Springs and Unnamed Hot Spring 1 are located in the Lower Geyser Basin and had somewhat alkaline pH (8.09 and 8.59) and hot temperatures (65.4°C and 84.5°C). These two hot springs had relatively high concentrations of fluoride ions, as well as chloride, sodium, and potassium. Palette Hot Spring was the only hot spring sampled in the Mammoth Hot Springs area of YNP and was the only calcium carbonate travertine depositing geothermal feature analyzed. Palette hot spring had an alkaline pH of 8.31, a cooler temperature of 25.8°C, and higher dissolved oxygen levels (6.7 mg/L) than other sites. Palette Springs had the highest levels of calcium, magnesium, silicon, and zinc of all sampled sites as well as high concentrations of sodium, potassium, and chloride. None of these more neutral/alkaline sites had a significant amount of dissolved heavy metals in their water (Table 2).

### 3.2 Culture independent microbial diversity analysis: taxonomy

#### 3.2.1 Kingdoms and eukaryotes

Taxonomic diversity was determined with 16S rRNA sequencing; the percentage distribution of kingdoms in the eight geothermal sites for water samples is shown in Supplementary Table 3; and in all sites, the majority of life in the water was from the Bacteria kingdom. Except for Sour Lake, the geothermal sites with very acidic pH values had a much larger percentage of Archaea than the more neutral and alkaline pH sites, whereas the higher temperatures correspond to a higher Archaea population. Viridiplantae, fungi, and eukaryote each made up less than 1.5% of the ASVs detected in all water samples. Similarly, there was a higher percentage of Archaea in sediment samples from acidic sites than in neutral or alkaline sites, except Sour Lake (Table 1). The eukaryotic diversity of all sites at the phylum and family taxonomy levels for the soil and water samples are shown in Supplementary Figures 1–4. In water samples, Ascomycota, Euglenida, Chlorophyta, and Streptophyta phyla were present in nearly all geothermal sites. Ascomycota dominated in Black Dragon’s Caldron and Streptophyta dominated in Emerald Springs—both of which had very low pH values. The most prevalent phylum in the soil samples were Chlorophyta, Streptophyta, Bacillariophyta, and Basidiomycota. Sour Lake soil samples had a predominance of Streptophyta phylum just like its water samples, whereas Chlorophyta dominated Black Dragon’s Caldron sediment. At the family taxonomic level, Black Dragon water primarily hosted *Hyaloscyphaceae* and *Euglenaceae* eukaryotes, while Emerald Springs primarily hosted *Solanaceae* and *Thoracosphaereaceae*. Black Dragon soil was primarily *Ericaceae* and *Dunaliellaceae*, and Sour Lake *Ericaceae* and *Hyalocyphaceae*. The less acidic geothermal sites also contained a significant amount of *Agaricaceae* and *Thalassiosisraceae* in Palette Springs. The predominant Eukarya families differed significantly not only between geothermal sites but also between the water and soil populations at the same site.

#### 3.2.2 Prokaryotes

**Figure 2:**
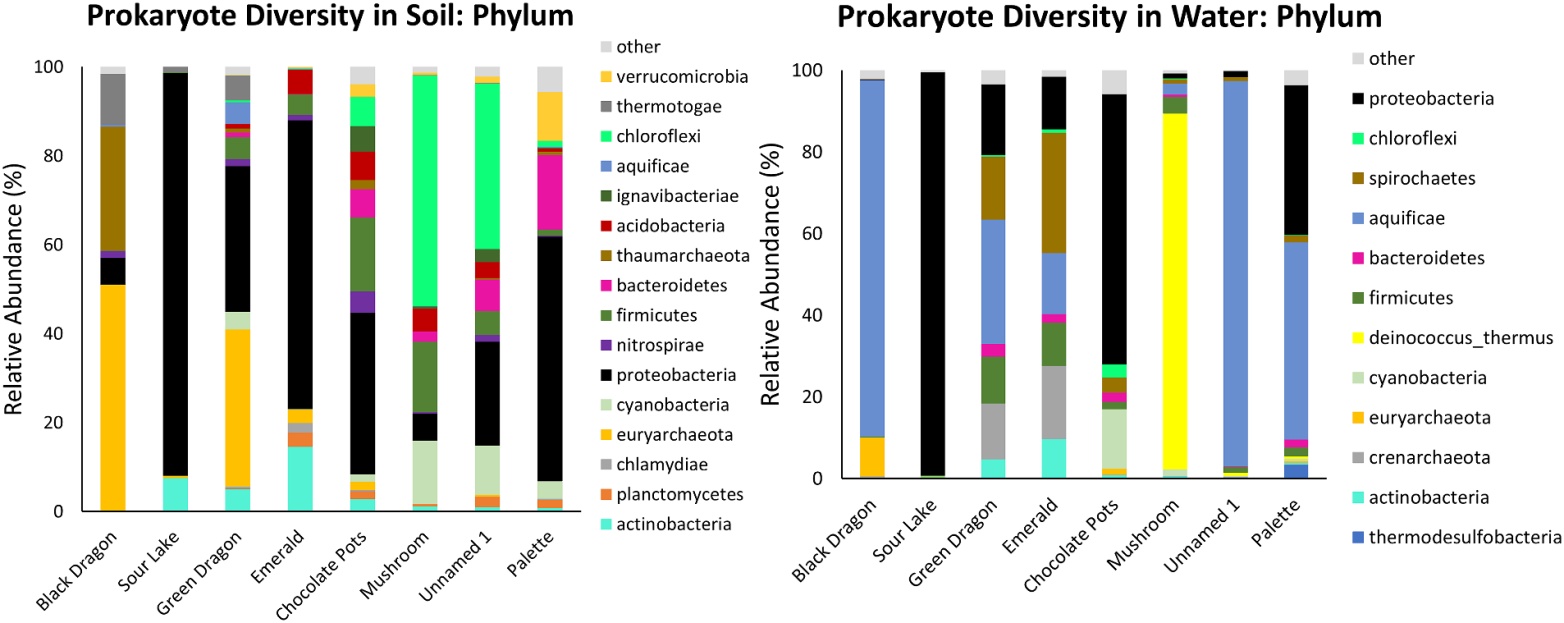
Prokaryotic taxonomic diversity of sediment and water samples at the phylum level. Only the top 10 phyla at each sample site that also has >2% abundance in the sample are shown.

**Figure 3:**
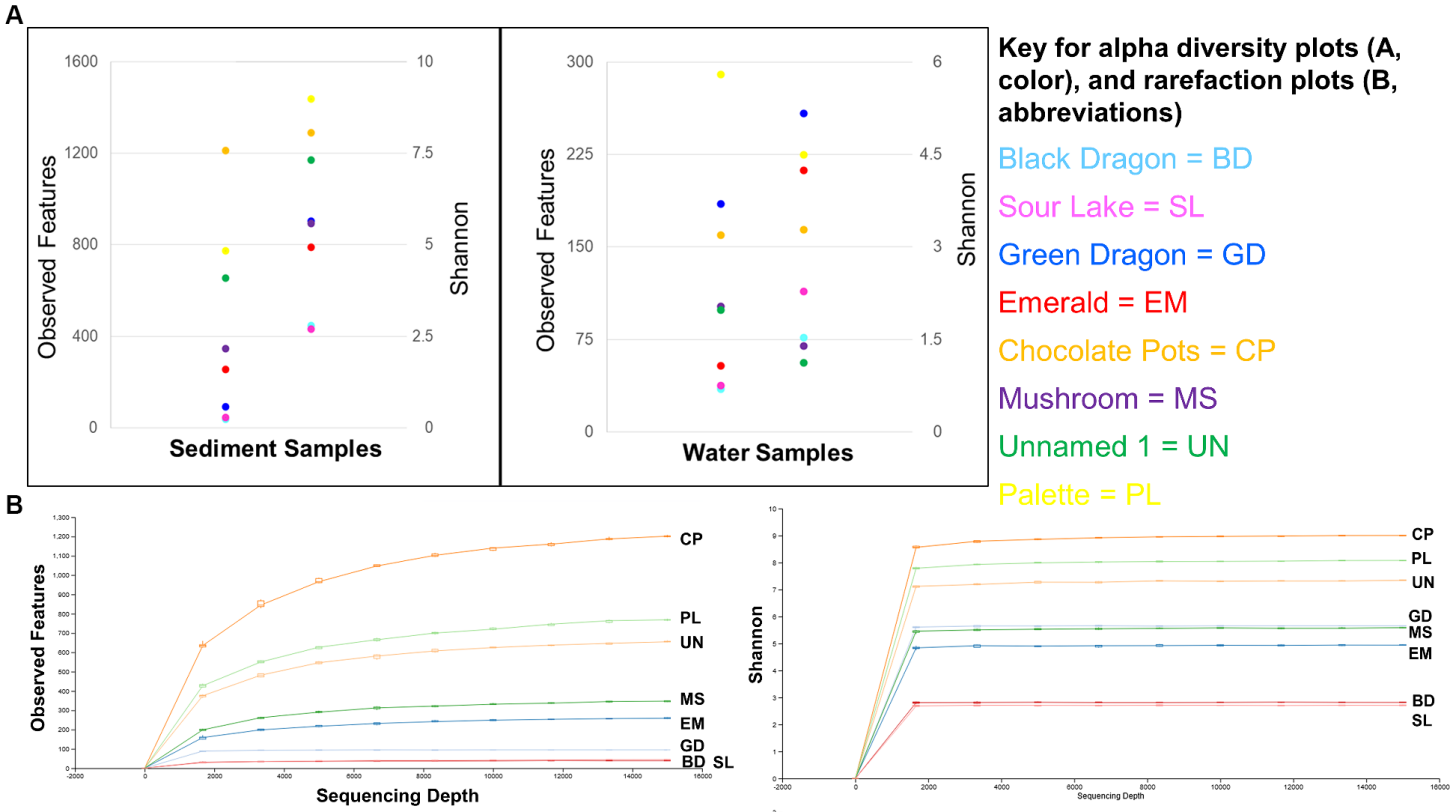
Alpha diversity: Observed features and Shannon’s entropy indices of sediment and water samples from all eight sites **(A)** and rarefaction curves for observed features and Shannon’s entropy for all sample sites **(B)**.

Prokaryote populations showed more diversity than eukaryote populations at all sites and were much more abundant as well (Table 1; Figure 2; Supplementary Table 3; Supplementary Figures 5, 6). Other than the common Proteobacteria the soil and water samples at the same site differed significantly in their phylum and family populations of prokaryotes, and the soil samples tended to have a greater diversity of prokaryotes than the water samples (Figures 2, 3; Supplementary Table 3; Supplementary Figures 5, 6). Most of the water samples also had significant populations of Aquificae, and Mushroom Springs was dominated by Deinococcus_thermus. At the family level, the acidic and hot geothermal waters contained significant amounts of *Aquificaceae*, although Sour Lake (acidic but not hot) predominantly hosted *Acidithiobacillaceae* and *Acetobacteraceae,* and Chocolate Pots had a large *Burkholderiaceae* population (Supplementary Figures 5, 6). Palette hot springs, which had the highest dissolved oxygen levels of any site, had the greatest prokaryotic diversity at the family level (Supplementary Figures 5, 6). The soil surrounding the acidic Black Dragon’s Caldron and Green Dragon Springs had a significant Euryarchaeota (Archaea) population, while the more alkaline sites of Chocolate Pots, Mushroom, and Unnamed Hot Spring 1 had a significant Chloroflexi population. The soil from more neutral to alkaline sites (especially Palette and Chocolate Pots) had significantly more diversity at the family level than the more acidic sites (Figure 2; Supplementary Figures 5, 6). Mushroom and Unnamed Hot Spring 1 had significant *Roseiflexaceae* family populations, while Green Dragon and Emerald had a significant population of *Rhodobacteraceae*. The soil next to the acidic sites had significant *Acetobacteraceae* and *Acidithiobacillaceae* family populations (Supplementary Figures 5, 6).

### 3.3 Alpha and beta microbial diversity

#### 3.3.1 Sediment and water samples alpha diversity

**Figure 4:**
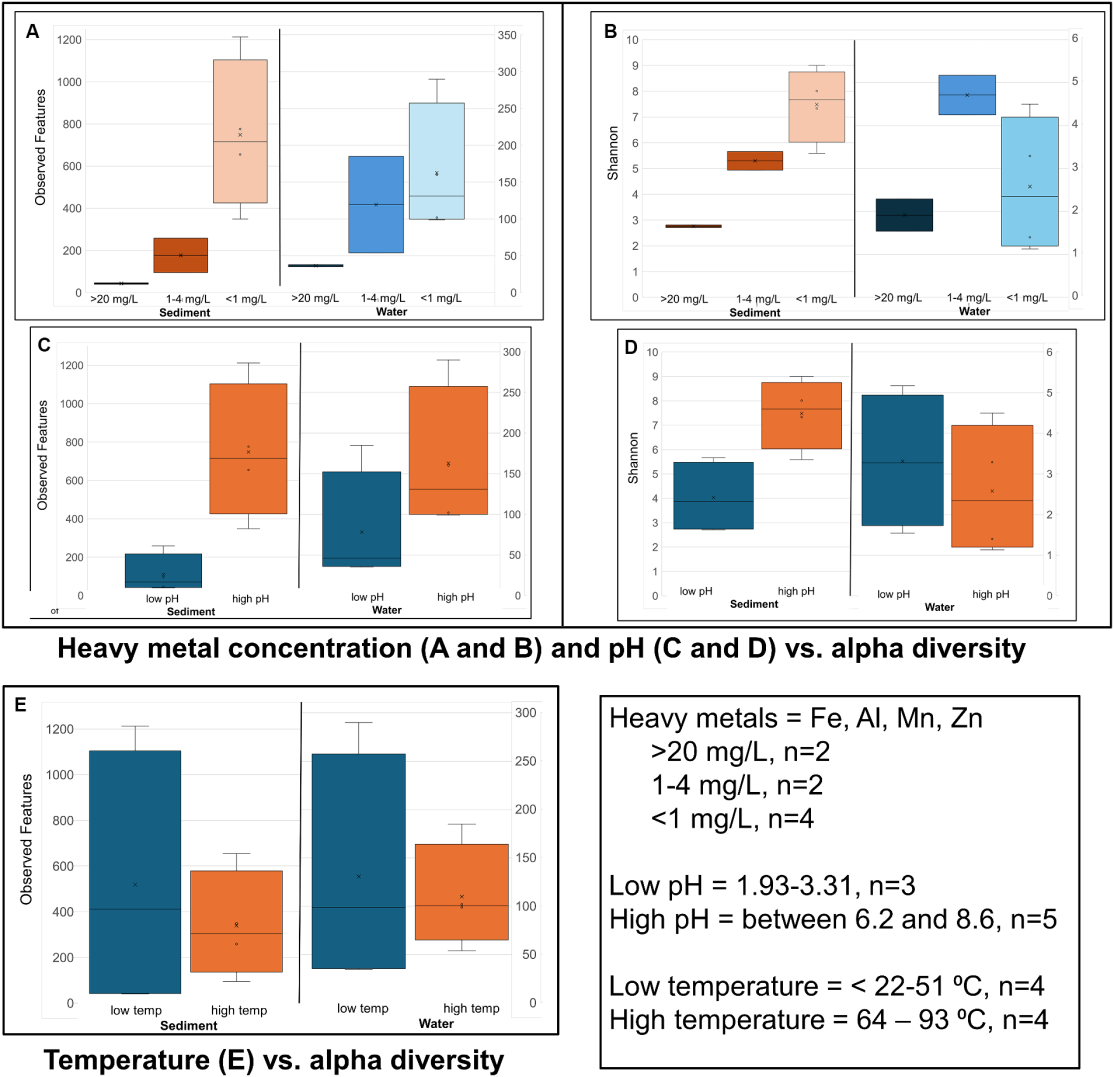
Alpha diversity: Box plots comparing alpha diversity (Observed features and Shannon’s) variation in sample sites that had high, relatively low, and near zero dissolved heavy metal concentration in water **(A,B)** sample sites with water of a low pH and neutral and slightly alkaline pH (high pH) **(C,D)**, and sample sites with water of a low vs. high temperature **(E)**.

The sediment samples were more diverse than the water samples (Supplementary Table 3) and showed a clear correlation to decreasing microbial diversity as the pH of the surrounding water decreased (Figure 4). A similar correlation to the surrounding water temperature was not observed (Figure 4).

**Figure 5:**
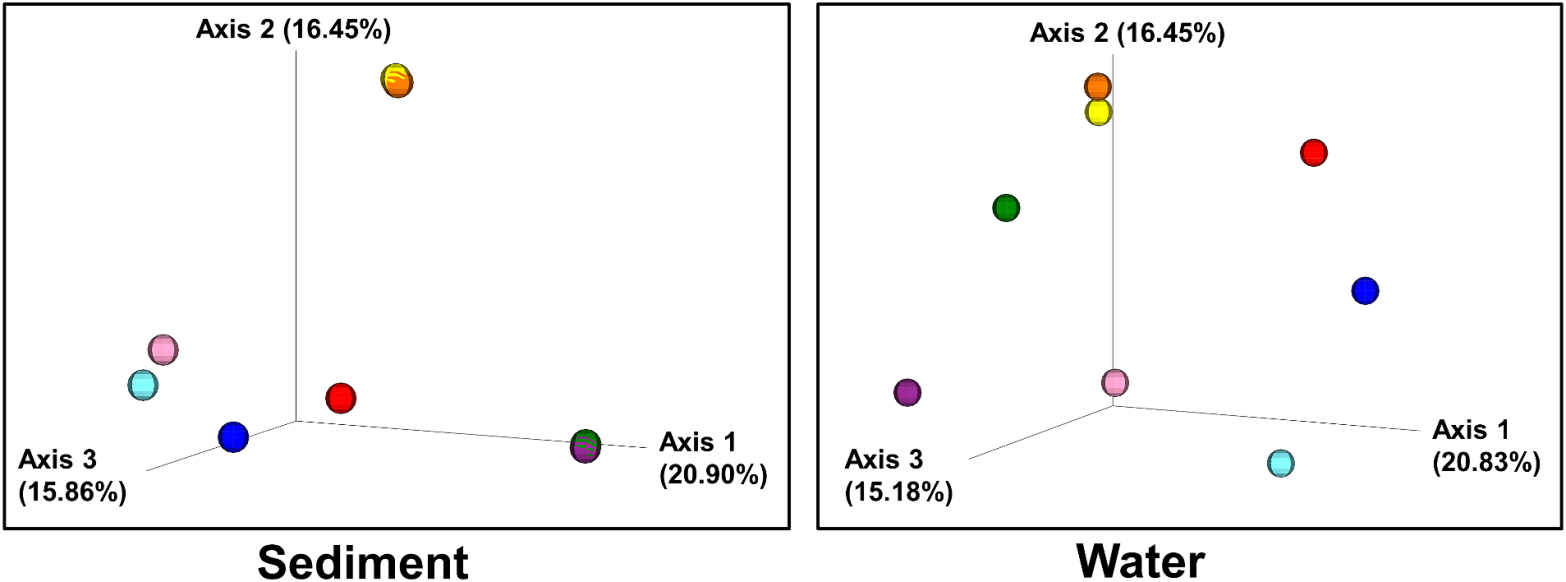
Beta diversity: Bray–Curtis dissimilarity of sites plotted in three-dimensional PCoA plots. Individual sites are color-coded:: 1(Black Dragon’s Caldron) light blue; 2 (Sour Lake) pink; 3 (Green Dragon) blue; 4 (Emerald) red; 5 (Chocolate Pots) orange; 6 (Mushroom) purple; 7 (Unnamed 1) green; 8 (Palette) yellow.

Alpha diversity box plots (Figure 4) comparing high and low pH sediment sites provided the following Kreskas–Wallis values: all groups: Faith and Observed features, H index = 5.83 and *p*-value = 0.0541; Shannon H = 4.764 and *p* = 0.0923. Pairwise Kruskal–Wallis values (high and low pH) = Faith and Observed Features: H = 4.5, *p* = 0.03389, q = 0.1017; Shannon: H = 3.125, *p* = 0.07710, q = 0.1797. Alpha diversity box plots (Figure 5) comparing high- and low-temperature sediment sites provided the following Kruskal–Wallis values: all groups and pairwise (high and low temperature): H index = 1.0888, *p*-value = 0.2967, q = 0.2967.

The alpha diversity of the water samples did not show straightforward relationship between either temperature or pH and decreasing alpha diversity.

Alpha diversity box plots (Figure 4) comparing high and low pH water samples provided the following Kruskal–Wallis values: all groups: Faith and Observed features, H index = 3.222, 2.097 and *p*-value = 0.19966, 0.3504, Shannon H = 1.0972 and *p* = 0.5777. Pairwise Kruskal–Wallis values (high and low pH) = Faith and Observed Features: H = 2.0, *p* = 0.1573, q = 0.2359, 0.4719; Shannon: H = 1.125, *p* = 0.2888, q = 0.8665. Alpha diversity box plots (Figure 4) comparing high- and low-temperature water samples provided the following Kruskal–Wallis values: all groups: Faith and Observed features, H index = 0.222, 0.5555 and *p*-value = 0.88149, 045605, Shannon H = 0.0222 and *p* = 0.8815. Pairwise Kruskal–Wallis values (high and low temperature) = Faith and Observed Features: H = 0.0222, 0.5555, *p* = 0.8815, 0.4560, q = 0.8815, 0.4560; Shannon: H = 0.0222, *p* = 0.8814, q = 0.8814.

#### 3.3.2 Sediment and water samples beta diversity

**Figure 6:**
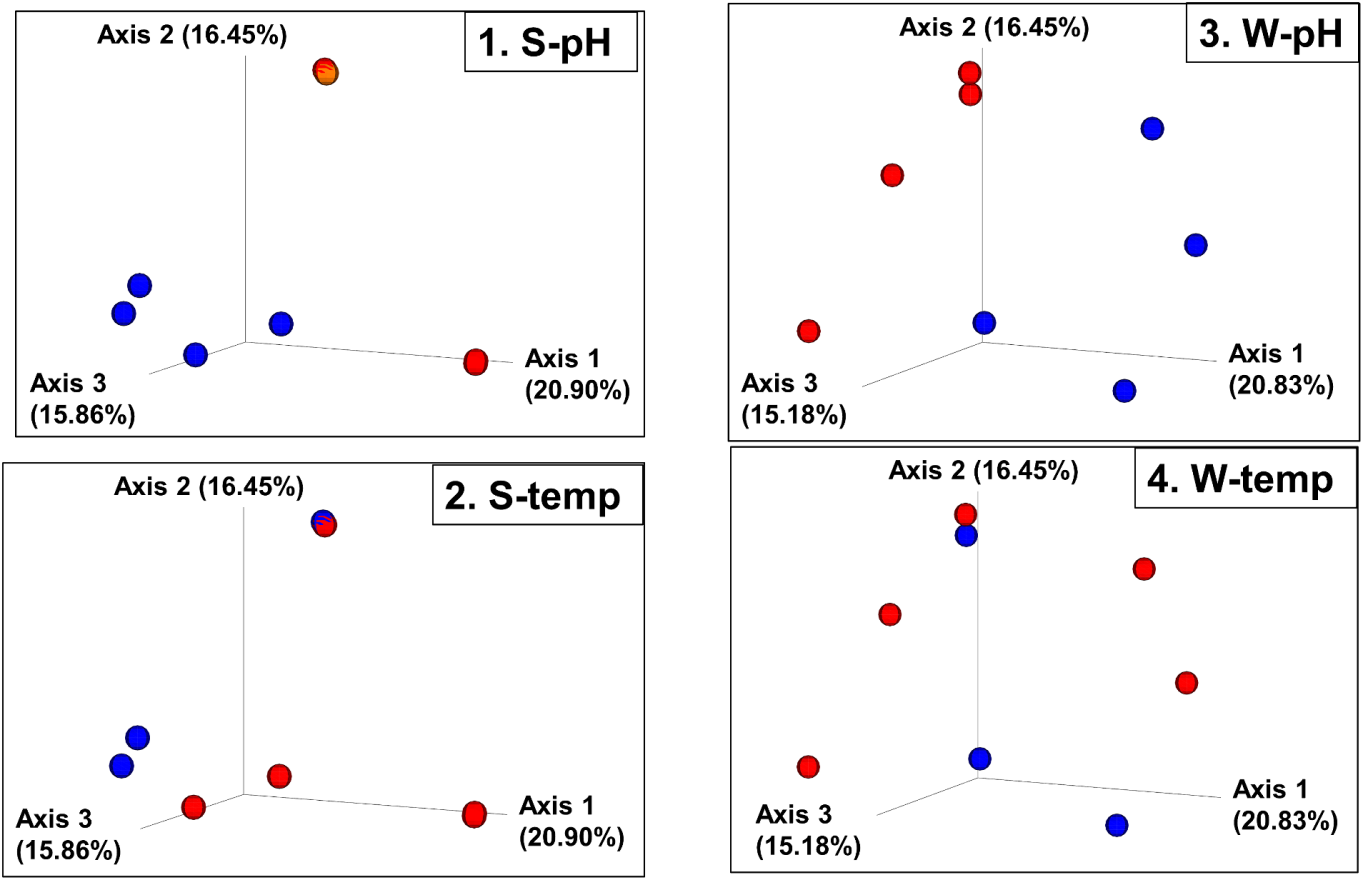
Beta diversity: Bray–Curtis dissimilarity PCoA plot with sites grouped into high and low pH and temperatures. (1. S-pH) and (3. W-pH) are sediment and water samples grouped into low pH (blue spheres, pH < 6) and higher pH (red spheres, pH > 8), respectively. (2. S-temp) and (4. W-temp) are sediment and water samples grouped into low temperature (blue spheres, temperature < 60°C) and high temperature (red spheres, temperature > 60°C), respectively.

Bray–Curtis Dissimilarity, Jaccard distance, Weighted and Unweighted UniFrac Indices of the sediment and water samples were plotted using principal coordinates analysis (PCoA) and plotted in three-dimensional Emperor PCoA plots. Individual samples are color-coded in Figure 5, and high and low pH samples and high- and low-temperature samples are indicated in red (high) and blue (low) spheres in Figure 6.

## 4 Discussion

The physicochemical parameters of hydrothermal fluids largely control which microbial populations are present in a hydrothermal feature (Colman et al., 2016; Narsing Rao et al., 2021; de la Haba et al., 2022; Shu and Huang, 2022). Previous studies have found that the primary physicochemical drivers in microbial populations within hydrothermal sites are temperature and pH, followed by dissolved solute distribution, including dissolved heavy metal concentration (Meyer-Dombard et al., 2005; Inskeep et al., 2015; Jiang, 2015; Colman et al., 2016; Jay et al., 2016; Jiang and Takacs-Vesbach, 2017; Hamilton et al., 2019; Bennett et al., 2020; Massello et al., 2020; Podar et al., 2020; Bennett et al., 2022; Peach et al., 2022). In YNP, the pH, temperature, and solute composition of hydrothermal waters are modulated by meteoric water (water from rain or snow-melt) mixing with subsurface thermal water brine exposed to magma gases, different rock compositions, and more (Parenteau and Cady, 2010).

Previous studies at YNP and around the world have indicated that not only can microbial populations vary significantly between geographically close hot springs but can also vary significantly within the same hot spring—both spatially and temporally (Schubotz et al., 2013; Wang et al., 2013, 2014; Colman et al., 2021; Bennett et al., 2022). Moreover, there is often a large difference between the water-associated and sediment-associated microbial populations within the same hot spring, and this ecological differentiation between planktonic and sediment-associated populations is also found globally in marine and freshwater systems (Colman et al., 2016; Zeng et al., 2019; Podar et al., 2020; Upin et al., 2023). This study compared the physicochemical parameters of all three types of hydrothermal fluids/features at YNP [acid sulfate (AS), alkaline chloride (AC), and calcium carbonate (CC)] and analyzed and compared their planktonic (water) and sediment (soil at the water interface) microbial communities using 16S rRNA sequencing. All samples in this study were collected within a 1-week period (reducing temporal variability) and subjected to identical collection, storage, DNA extraction, and sequencing methods. This homogeneity in collection and analysis methods significantly decreases variables that are typically present when comparing microbial diversity at different sites and analyzing the effect of physicochemical parameters on the extent of diversity and microbial population profile.

### 4.1 Taxonomy

#### 4.1.1 Sediment prokaryotes

Similar to previous studies, a significantly different population of prokaryotes dominated the sediment samples from the YNP geothermal sites as compared to the water counterparts, with the soil microbial community appearing more diverse than their bordering water communities (Figure 3; Colman et al., 2016). In terms of Archaea, Black Dragon’s Caldron, Green Dragon, and Emerald Springs all had a significant Euryarchaeota population, with Chocolate Pots and Sour Lake having a lower, but still a significant relative abundance (Table 1; Figure 2). The soil surrounding Black Dragon’s Caldron was unique in that its phylum-level prokaryote population was primarily Archaea (78% relative abundance), with 51% of its population being from Euryarchaeota and 28% from Thaumarchaeota. Black Dragon’s Caldron is also unique in that it is a relatively new geothermal site, the mud pot having bubbled out of a crack in the Earth in 1948 (Smith and Siegel, 2000). This warm and acidic mud pot also had very high conductivity and levels of sulfate ions and heavy metals (Fe, Al, Mn, and Zn) compared to the other sites (Table 2; Supplementary Table 2). To the best of our knowledge, this is the first microbial diversity study looking at soil or water at Black Dragon’s Caldron (as well as Unnamed Hot Spring 1), although a nearby hot spring (Jim’s Black Pool) with a similarly high iron concentration was also found to have an unusually high and diverse archaea population (Barns et al., 1994).

Proteobacteria, which is commonly abundant in soil, was prevalent in all the soil samples, with Sour Lake being over 90% Proteobacteria (Spain et al., 2009). The three sites with the most acidic water, Black Dragon’s Caldron, Sour Lake, and Green Dragon Springs, had soil with a significant amount of the phylum Thermotogae while all sites except Black Dragon’s Caldron had between 1 and 17% relative abundance of Firmicutes. Thermotogae are mostly thermophilic and hyperthermophilic bacteria and all sites where Thermotogae was in abundance had waters with a temperature over 50°C, except Sour Lake (Conners et al., 2006). Globally, Firmicutes are commonly found in hot spring environments (Poddar and Das, 2018; Saghatelyan et al., 2021; Kochetkova et al., 2022). The more neutral and alkaline sites all had notable amounts of Chloroflexi, Bacteroidetes, and Cyanobacteria. Other studies have also shown a large relative abundance of Chloroflexi and Cyanobacteria in more alkaline hot spring environments, with the richness of Cyanobacteria decreasing with increasing temperature while Chloroflexi richness not significantly changing with increasing temperature (Bennett et al., 2020). The relative abundance of Chloroflexi and Cyanobacteria phylum did not correlate with temperature (or pH) in the sites we studied, although no significant amount of Chloroflexi was found below a pH of 6.2 or above a temperature of 84°C.

#### 4.1.2 Water prokaryotes

Globally, various studies have found that Aquificae, Proteobacteria, and Crenarchaeota often dominate hot springs waters (Shu and Huang, 2022). Our taxonomic analysis of the phylum level of the water samples confirmed these findings for Proteobacteria and Aquificae (Figure 2), but only Emerald and Green Dragon hot springs had significantly high populations of the Archaea Crenarchaeota (these two sites also had a large population of Spirochaetes bacteria). Euryarchaeota was the predominant Archaea phylum in Black Dragon’s Caldron and Chocolate Pots (Figure 2). Except for Chocolate Pots, all water sites with a significant percentage of Archaea present had an acidic pH and temperatures greater than 48°C. The single water sample outlier that did not follow the trend of having a very high proportion of Aquificae and/or Proteobacteria was Mushroom Spring—in which Deinococcus-thermus predominated, *Thermacecea* family especially. Although our sequencing did not give reliable species-level differentiation, a likely candidate for such a high level of *Thermacecea* is the thermophilic bacteria *Thermus aquaticus*, which was discovered in 1969 by Brock in Mushroom Spring and led to the isolation of Taq DNA polymerase (Brock, 2012). Sour Lake and Chocolate Pots did not contain a significant amount of Aquificae, even though Sour Lake had a very low pH and all other low pH sites contained a significant population of Aquificae. Instead, the water in Sour Lake was primarily composed of Proteobacteria, specifically *Acidithiobacillaceae* and *Acetobacteraceae* families (which were also abundant in the Sour Lake sediment sample). The very low, acidic pH of Sour Lake is caused by *Acidithiobacillaceae* bacteria in the lake metabolizing the high levels of sulfur in the area to form sulfuric acid (Johnson and Aguilera, 2016). Similarly, the species of *Acetobacteraceae* present are likely acidophilic, as these are known to inhabit acidic hot springs (Saini et al., 2021).

### 4.2 Alpha and beta microbial diversity

#### 4.2.1 Sediment alpha diversity

The sediment samples from sites with acidic pH had significantly less diversity than the more neutral or slightly alkaline sites in all alpha diversity metrics measures: Observed features, Faith and Shannon indexes. Chocolate Pots and Palette Springs had the greatest alpha diversity of the sites studied with both sites having water pH values closer to neutral, mild temperatures, and low to zero concentration of dissolved heavy metals. For all three alpha diversity metrics, the trends of decreasing pH and increasing dissolved heavy metal concentration of water the soil was exposed to corresponded to lower diversity numbers (Figures 3, 4; Supplementary Table 3). Some exceptions included Chocolate Pots, with a pH of 6.2 and near zero heavy metals dissolved, having higher alpha diversity than any of the other more alkaline sites, and Palette having higher diversity than the Unnamed Hot Spring 1. Importantly, both of these sites (Chocolate Pots and Palette) were very shallow sites in which samples had to be collected within a few centimeters of the surface. Additionally, Shannon’s showed a slightly different pattern (Figure 3), although the low pH and high dissolved heavy metal concentration sites having lower alpha diversity trend remained.

However, a similar alpha diversity correlation in the sediment did not hold with increasing or decreasing temperature of the surrounding water (Figure 4). Similarly, there was not a clear correlation between (non-heavy) dissolved metal concentrations (Na, K, Mg, and Ca), total ion concentrations (NH_4_^+^, F^−^, Cl^−^, NO_3_^−^, HPO_4_^−^, and SO_4_^2−^), and dissolved H_2_S or chloride gas of the sample site water and increases or decreases in alpha diversity in the sediment (Table 2; Supplementary Tables 1–3).

#### 4.2.2 Water alpha diversity

There was a correlation, but not as strong of one, between decreasing pH and increasing dissolved heavy metal concentrations resulting in decreasing alpha diversity in the water samples (Figure 4; Supplementary Table 3). Although lower pH levels somewhat corresponded to lower alpha diversity metrics, the trend had many exceptions. For example, Green Dragon Spring had much higher Faith, Observed Features, and Shannon index values than Mushroom Springs even though Green Dragon Spring had a pH = 2.93 and a temperature of 93°C, whereas Mushroom Spring had a pH of 8.09 and a temperature of 64°C. Additionally, the comparison of Shannon’s and Faith indices, which shows diversity in terms of richness and evenness of low and high pH groups of water samples, did not show a pH correlation, although the richness only Observed features metric did (Figure 4; Supplementary Table 3). This suggests that the decreasing pH values in water affect evenness more than richness. High levels of dissolved heavy metals also correlated to low alpha diversity metrics, with Black Dragon’s Caldron and Sour Lake (overall heavy metal concentrations of 86 and 24 mg of heavy metals/L, respectively) having much lower alpha diversity metrics than all other sample sites. The two sites with the next highest levels of dissolved heavy metals, Emerald Springs (1.19 mg/L) also had relatively low diversity metrics, but Green Dragon springs had significantly higher diversity than Emerald even though it had a slightly higher heavy metal concentration (2.26 mg/L), a similarly low pH and the highest temperature of all sites (93°C). No clear explanation for Green Dragon’s unusually high alpha diversity was found in the data or literature. Similar to the pH trend, the richness (Observed features) of the microbial community showed a much stronger correlation to dissolved heavy metal concentration than diversity richness and diversity-weighted metrics (Faith and Shannon). In addition, similar to the sediment samples, no clear trend in increasing temperature and other non-heavy metal dissolved solute concentrations and alpha diversity metrics were observed during analysis (Figure 4; Supplementary Tables 1–3).

#### 4.2.3 Sediment and water beta diversity

The beta diversity (the measure of similarity or dissimilarity, in terms of types of organisms present, in two community populations) of the sediment microbial populations among different hot springs also showed a stronger pH association than temperature (Figures 5, 6; Supplementary Figures 7, 8), although the low- and high-temperature groups also clustered in different regions in the PCoA plot. The eight sample sites were grouped into high and low pH groups (red and blue spheres in Figure 6) and high- and low-temperature groups (red and blue spheres in Figure 6), and Bray–Curtis dissimilarity, Jaccard Distance, Weighted, and Unweighted UniFrac Indices of the water samples were plotted using principal coordinates analysis (PCoA) and plotted in three-dimensional PCoA Emperor plots (Figures 5, 6; Supplementary Figures 7–10). There is a clearer differential clustering of the high pH sites than the low pH sites in all four beta diversity metric UniFrac plots (Liu et al., 2021). The concomitant samples grouped by high vs. low temperature also showed differential clustering of the low and high temperature sites, but not as clearly as the pH groups.

In terms of beta diversity of water samples, the Bray–Curtis and Unweighted UniFrac PCoA plots of the water samples showed clear clustering of beta diversity based on low or high pH (Figures 5, 6; Supplementary Figures 7–10) in the water samples, but not as clear clustering in the low- and high-temperature groups.

Previous studies, such as the study of Colman et al. (2016), analyzed the physicochemical parameters and microbial diversity profiles of 15 different hot springs in YNP, differentiating between planktonic and sediment populations and their data indicated that although water microbial populations are primarily driven by temperature and pH, sediment populations are driven more by the availability of dissolved and mineral substrates capable of supporting microbial metabolism. Our results do not support this generalization; however, their study focused only on non-photosynthetic hot springs in YNP, which are hot springs with a temperature greater than 73°C, whereas our sampling sites had a larger temperature range that included photosynthetic and non-photosynthetic capable sites. Several studies have also reported temperature as the strongest factor shaping microbial community composition in hot springs, while other studies indicate that pH is the stronger driver (Jones et al., 2009; Rousk et al., 2010; Xiong et al., 2012; Kuang et al., 2013; Sharp et al., 2014; Zhalnina et al., 2015; Teng et al., 2017; Podar et al., 2020). Specifically, a massive 16S rRNA dataset analysis of 925 individual hot springs from the Taupo Volcanic Zone in New Zealand indicates that temperature only has a significant effect on diversity above 70°C, below which pH is the primary driver (Power et al., 2018). Similarly, a recent global study of microbial diversity in hot springs from around the globe also indicated that pH, along with SO_4_^2−^ concentration and abundances of certain phyla, is moderately correlated with microbial diversity, although most microbial variance remains unexplained (Hamilton et al., 2019; Barbosa et al., 2023). A challenge when comparing results from multiple different studies, however, is that there are a lot of different biases in different studies, especially in analytical chemistry and nucleic acid extraction methods and amplification protocols in 16S rRNA sequencing (Lauber et al., 2010, 2013; Hinlo et al., 2017; Colman et al., 2021).

Our 16S rRNA analysis of the different YNP hot springs indicates that water pH is a primary driver of alpha and beta prokaryotic microbial diversity in the soil that is regularly exposed to that water when geothermal sites with a wide range of pH and temperature are compared. There is a less strong, but still noticeable, correlation of the pH of the water with the alpha and beta diversity of the prokaryotic microbial population in the water. Similarly, higher dissolved heavy metal concentrations in water led to significantly less alpha diversity in both the sediment and water. Metal salt solubility noticeably increases with lower pH such that the low pH sites (with pH values below 3) are expected to have higher concentrations of dissolved metals than the more neutral or slightly alkaline site, as metal salt solubility tends to be at a minimum at a pH of approximately 8. However, the observation that the two sample sites with very high heavy metal concentrations (Black Dragon’s Caldron and Sour Lake, 86 and 24 mg/L of dissolved Fe, Al, Mn, and Zn, respectively, with pH values of 1.93 and 2.15) had much lower alpha diversity metrics than the other 2 very low pH sample sites (Green Dragon and Emerald, pH = 2.93 and 3.31) that had much lower heavy metal concentrations (1–2 mg/L) suggests that high levels of dissolved heavy metals can strongly decrease the alpha diversity of both sediment and water. Increasing temperature and the concentration of non-heavy metal dissolved solutes did not correlate strongly with alpha diversity in the sediment or water samples, but the temperature did seem to affect beta diversity.

In conclusion, eight sites in YNP were collected and analyzed to assess the physicochemical and microbial characteristics of both the water and the soil that is immediately, or intermittently, exposed to the geothermal water. To the best of our knowledge, this is the first report of the microbial diversity of Unnamed Hot Spring 1 and Black Dragon’s Caldron—a bubbling mud pot that was formed in 1948 and has an unusually high concentration of iron and archaea population. This study is novel in that all sampling sites had identical sampling, analytical and nucleic acid extraction methods, and amplification protocols—significantly reducing potentially biasing variables, and a wide range of hot springs with different pH levels, temperatures, and dissolved solutes were simultaneously compared. The pH and dissolved heavy metal concentrations of the water at the sample sites were highly correlated to alpha and beta diversity of the sites, with the temperature being less influential.

## Data availability statement

The datasets presented in this study can be found in online repositories. The names of the repository/repositories and accession number(s) can be found in the article/Supplementary material.

## Author contributions

LR: Conceptualization, Data curation, Formal analysis, Funding acquisition, Investigation, Methodology, Project administration, Resources, Supervision, Visualization, Writing – original draft, Writing – review & editing. SD: Formal analysis, Investigation, Writing – original draft, Conceptualization, Data curation, Methodology, Resources. KD: Formal analysis, Investigation, Writing – original draft. CK: Formal analysis, Investigation, Writing – original draft. MV: Formal analysis, Investigation, Writing – original draft. AJ: Formal analysis, Investigation, Writing – original draft. CT: Formal analysis, Investigation, Writing – original draft.
