# Supplementary material for: Comparing microbial populations from diverse hydrothermal features in Yellowstone National Park: hot springs and mud volcanoes
Source: Front Microbiol. 2024 Jun 27;15:1409664. doi: 10.3389/fmicb.2024.1409664 (PMC11236564; doi:10.3389/fmicb.2024.1409664)
Supplement: Supplementary file 1 [file Data_Sheet_1.DOCX]

Supplementary Material

1.1. Accession Numbers for Sequencing Data in Paper: Submitted as SRA Data to NCBI.

| PRJNA1094411 | Yellowstone National Park hot springs and mud volcano microbial diversity, Mar 27 '24 |
| --- | --- |

| Geothermal Site | Accession Numbers  (sediment, water) |
| --- | --- |
| Emerald Springs | SAMN40695429, SAMN40695430 |
| Green Dragon Springs | SAMN40695431, SAMN40695432 |
| Chocolate Pots Springs | SAMN40695433, SAMN40695434 |
| Unknown Hot Spring #1 | SAMN40695435, SAMN40695436 |
| Mushroom Springs | SAMN40695437, SAMN40695438 |
| Palette Springs | SAMN40695439, SAMN40695440 |
| Black Dragons Caldron Mud Volcano | SAMN40695441, SAMN40695442 |
| Sour Lake | SAMN40695443, SAMN40695444 |

## Supplementary Figures and Tables

**Table S1:** Physicochemical parameters of geothermal sites determined in the field at time of collection. Temperature was determined with an immersed probe, and O_2_, Cl_2_, and H_2_S are dissolved gas concentrations at the time of sampling. Black Dragons Caldron contained too much sediment for colorimetric gas measurements (ND=not determined) and Unnamed 1 and Palette Springs had no detectable H_2_S and Cl_2_, respectively. Conductivity reported from previous literature indicated the conductivity (from top to bottom of table) of the sites are: 8600, 6000, 1522, 2600, 743*, 1310, ND (but nearby Octopus Spring = 1459), and 2400 µS/cm (conductivity data from Yellowstone National Park Research Coordination Network, Montana State University, <http://rcn.montana.edu/> and *2009 USGS Water Chemistry Survey of Selected Spring, Geysers, and Streams YNP, <https://d9-wret.s3.us-west-2.amazonaws.com/assets/palladium/production/s3fs-public/atoms/files/Sample-Information_0.pdf>)

| Name | pH | Temp.  (°C) | O_2_  (mg/L) | Cl_2_  (mg/L) | H_2_S  (mg/L) |
| --- | --- | --- | --- | --- | --- |
| Black Dragon | 1.93 ± 0.03 | 48.0 ± 0.4 | ND | ND | ND |
| Sour Lake | 2.15 ± 0.03 | 21.7 ± 1.6 | 2.3 ± 0.4 | 0.01 ± 0.01 | 0.13 ± 0.01 |
| Green Dragon | 2.93 ± 0.01 | 93.0 ± 1.0 | 2.4 ± 0.5 | 0.01 ± 0.01 | 0.25 ± 0.04 |
| Emerald | 3.31 ± 0.02 | 77.6 + 2.6 | 1.9 ± 0.4 | 0.04 + 0.02 | 0.03 ± 0.01 |
| Chocolate Pots | 6.20 ± 0.06 | 50.8 + 0.6 | 2.1 ± 0.1 | 0.01 ± 0.01 | 0.03 ± 0.02 |
| Mushroom | 8.09 ± 0.02 | 64.5 + 0.3 | 2.7 ± 1.9 | 0.04 ± 0.00 | 0.02 + 0.01 |
| Unnamed 1 | 8.59 ± 0.04 | 84.5 + 0.9 | 1.0 ± 0.1 | 0.12 ± 0.08 | - |
| Palette | 8.31 ± 0.02 | 25.8 + 0.1 | 6.7 ± 0.2 | - | 0.02 + 0.00 |

**Table S2:** Select ion concentration of geothermal sites as determined by ion chromatography.

| Name | NH_4_^+^  (mg/L) | F^-^  (mg/L) | Cl^-^  (mg/L) | NO_3_^-^  (mg/L) | HPO_4_^-^  (mg/L) | SO_4_^2-^  (mg/L) |
| --- | --- | --- | --- | --- | --- | --- |
| Black Dragon | 3.11 | 1.37 | 33.20 | 0.07 | - | 1028.86 |
| Sour Lake | 1.78 | 0.73 | 99.42 | - | - | 428.07 |
| Green Dragon | 0.73 | 2.41 | 156.18 | 0.01 | - | 74.95 |
| Emerald | 0.94 | 3.11 | 332.00 | - | - | 65.960 |
| Chocolate Pots | 0.03 | 2.15 | 12.56 | - | - | 10.22 |
| Mushroom | - | 9.43 | 107.20 | 0.01 | 0.03 | 10.02 |
| Unnamed 1 | 0.78 | 12.74 | 112.66 | 0.22 | - | 8.95 |
| Palette | 3.11 | 0.87 | 88.84 | - | - | 284.00 |

**Table S3:** Alpha diversity metrics of soil samples (top, black) and water samples (bottom, blue) from the geothermal sites. Temperature and pH reported is that of the water at the geothermal site.

| Name | pH | Temperature  °C | Faith | Observed Features | Shannon |
| --- | --- | --- | --- | --- | --- |
| Black Dragon | 1.93 | 48.0 | 8.33  8.51 | 40  35 | 2.81  1.54 |
| Sour Lake | 2.15 | 21.7 | 13.82  11.03 | 46  38 | 2.71  2.29 |
| Green Dragon | 2.93 | 93.0 | 15.39  29.62 | 95  185 | 5.66  5.17 |
| Emerald | 3.31 | 77.6 | 37.40  8.04 | 258  54 | 4.94  4.25 |
| Chocolate Pots | 6.20 | 50.8 | 144.99  32.47 | 1213  160 | 9.00  3.29 |
| Mushroom | 8.09 | 64.5 | 50.71  22.33 | 349  102 | 5.59  1.40 |
| Unnamed 1 | 8.59 | 84.5 | 82.64  16.06 | 655  99 | 7.33  1.13 |
| Palette | 8.31 | 25.8 | 99.93  55.18 | 776  290 | 8.07  4.50 |


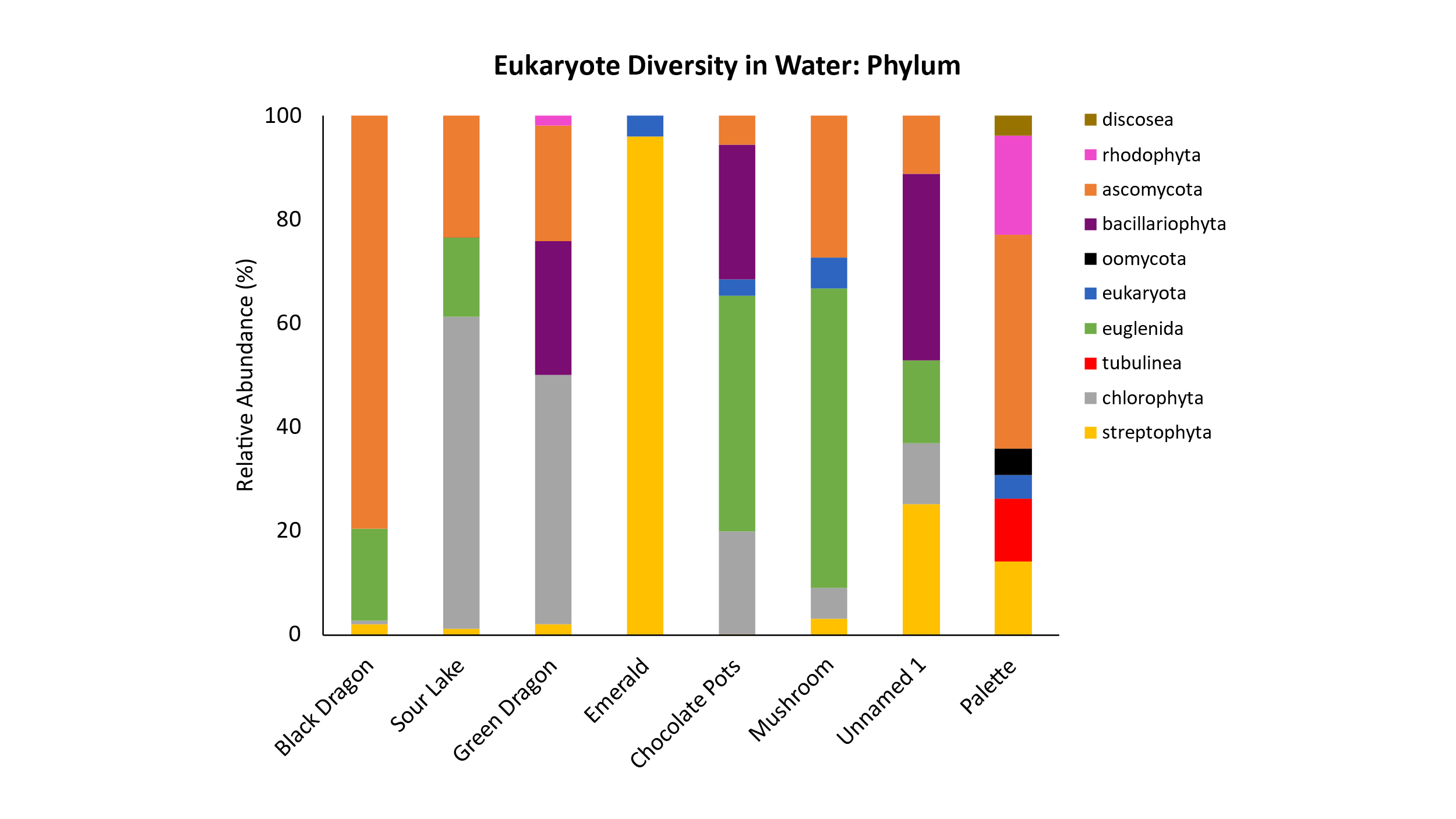


**Supplementary Figure 1.** Eukaryotic water taxonomic diversity at the phylum level. Only the top 10 phylum at each sample site that also have >2% abundance in the sample are shown.


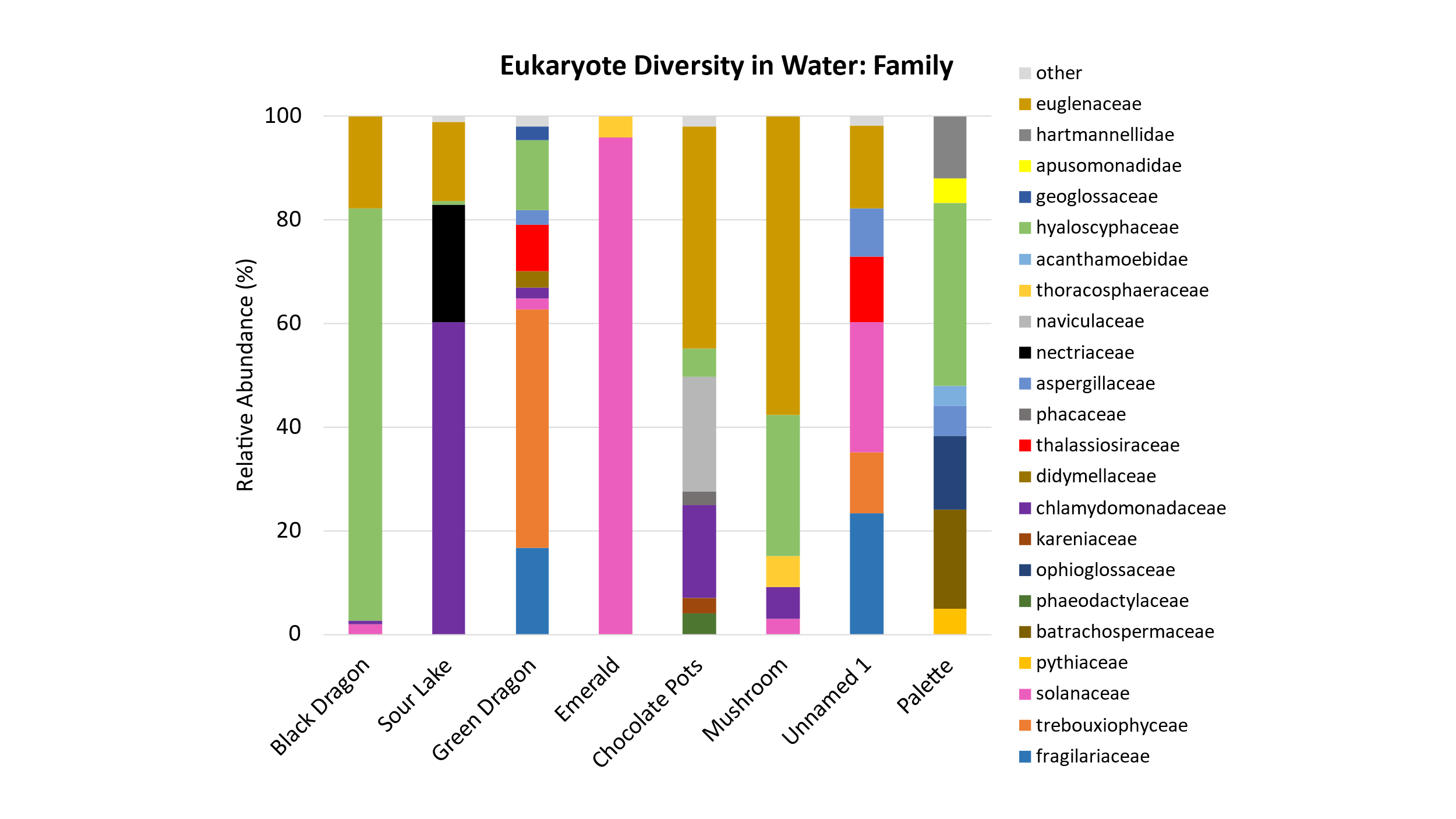


**Supplementary Figure 2.** Eukaryotic water taxonomic diversity at the family level. Only the top 10 families at each sample site that also have >2% abundance in the sample are shown.


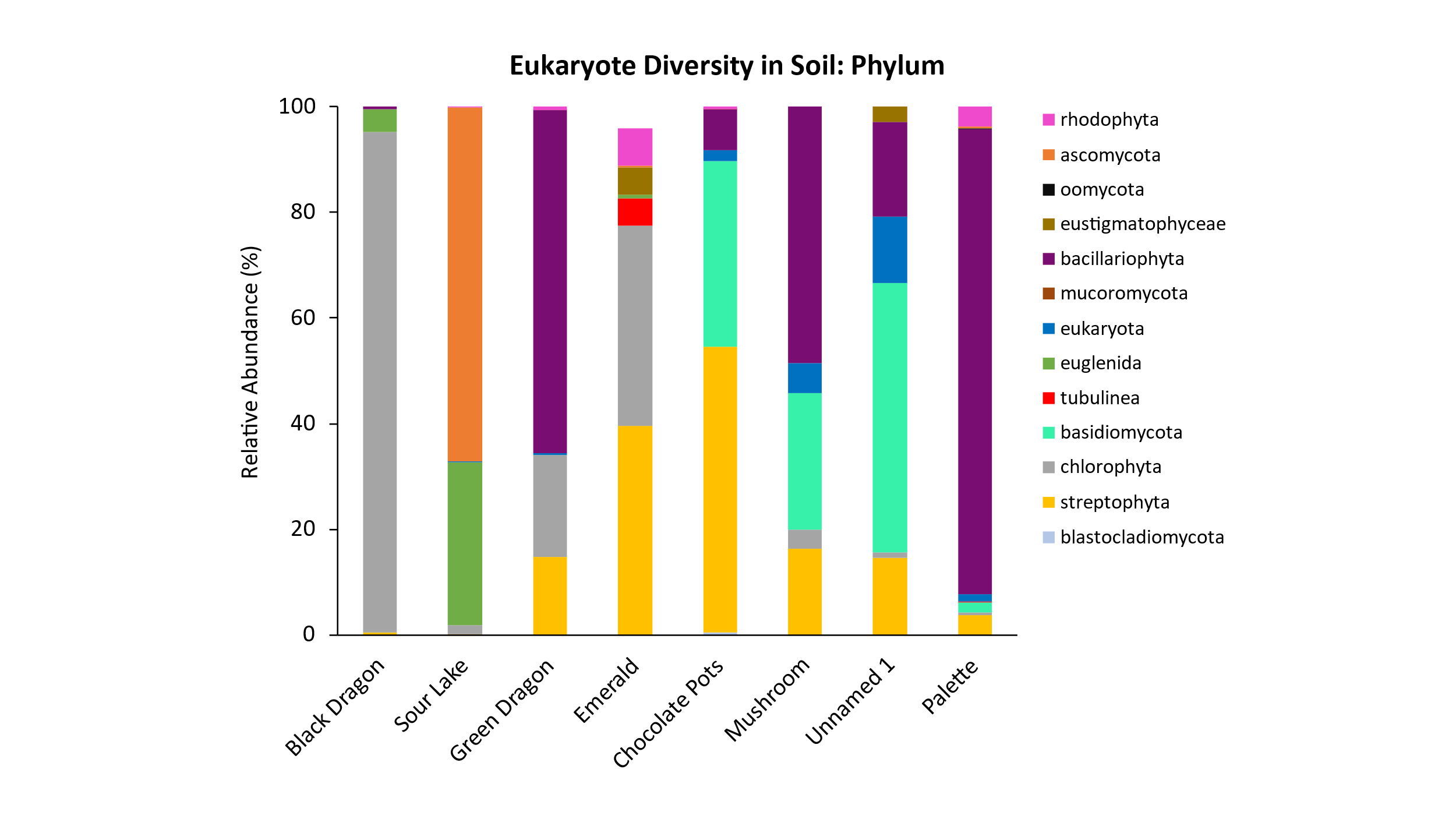


**Supplementary Figure 3.** Eukaryotic sediment taxonomic diversity at the phylum level. Only the top 10 phylum at each sample site that also have >2% abundance in the sample are shown.


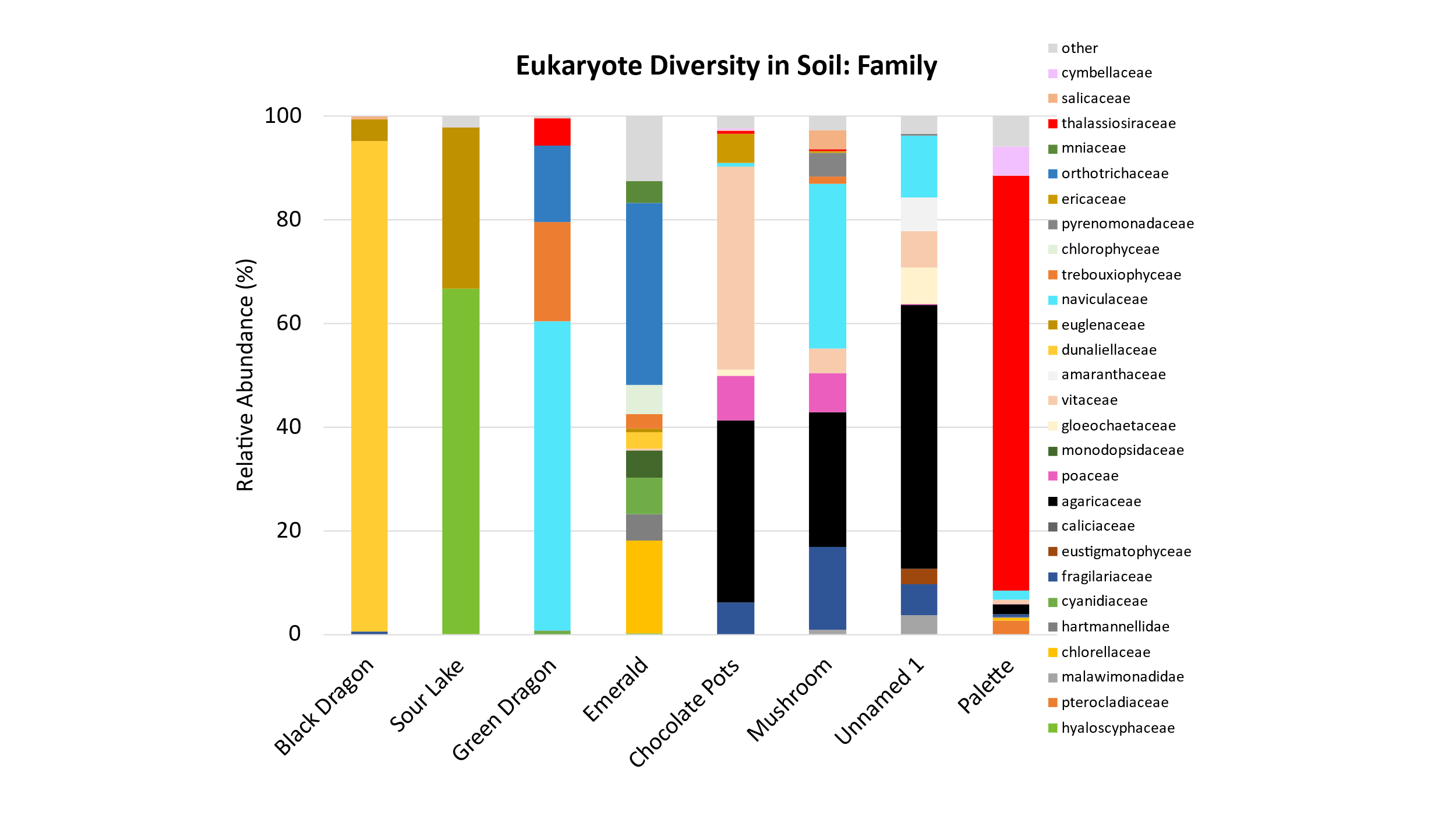
**Supplementary Figure 4.** Eukaryotic sediment taxonomic diversity at the family level. Only the top 10 families at each sample site that also have >2% abundance in the sample are shown.


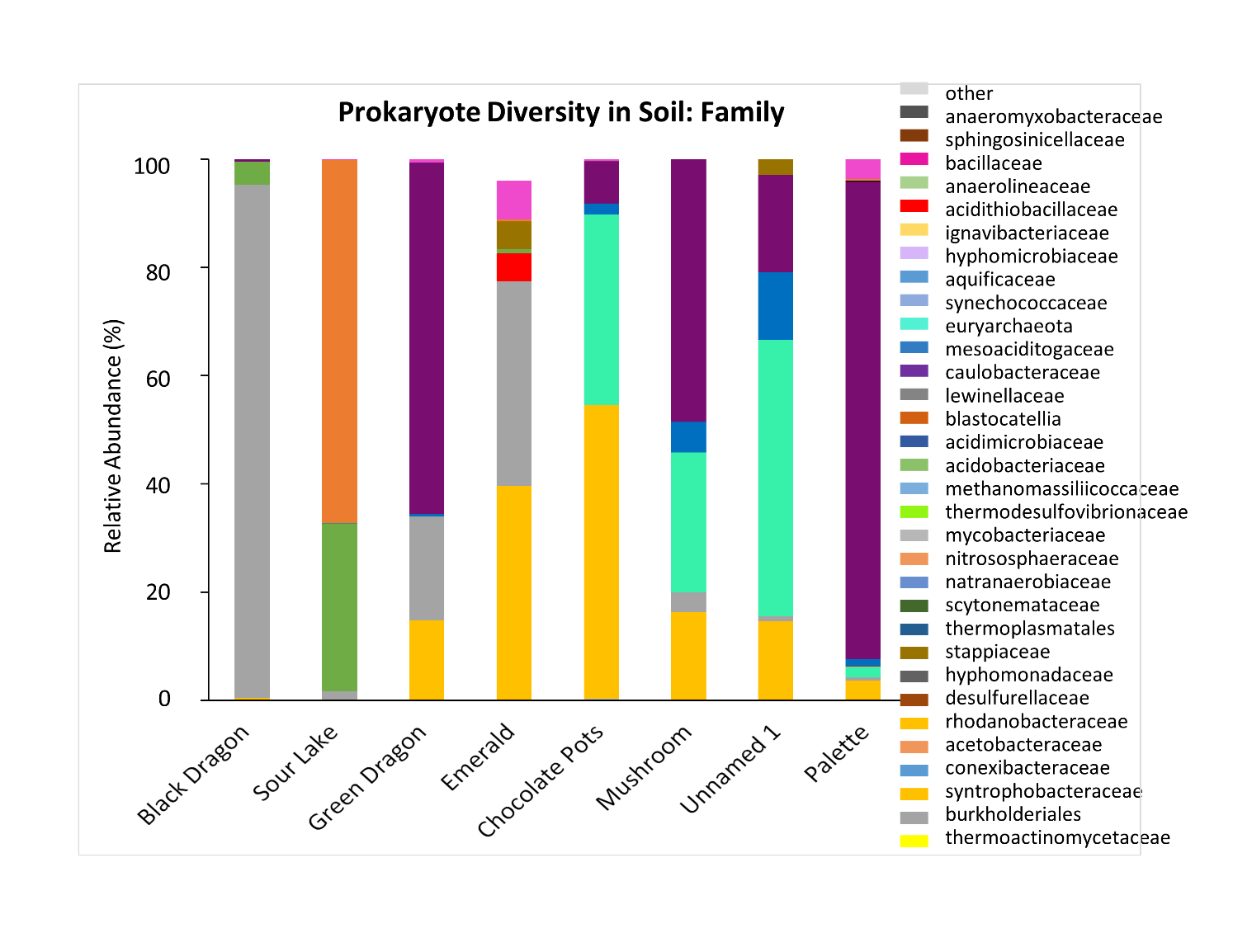


**Supplementary Figure 5.** Prokaryotic sediment taxonomic diversity at the family level. Only the top 10 families at each sample site that also have >2% abundance in the sample are shown.


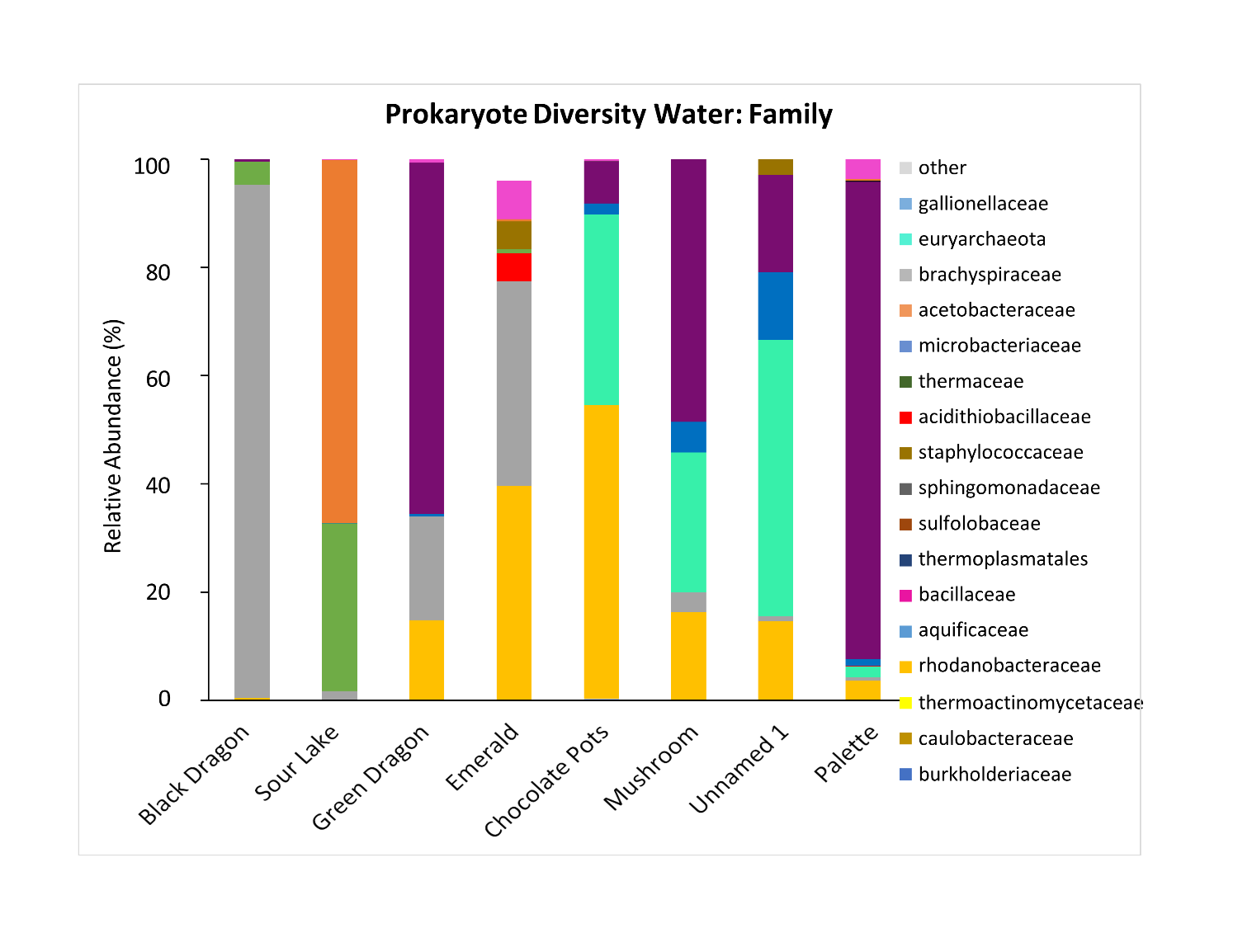


**Supplementary Figure 6.** Prokaryotic water taxonomic diversity at the family level. Only the top 10 families at each sample site that also have >2% abundance in the sample are shown.


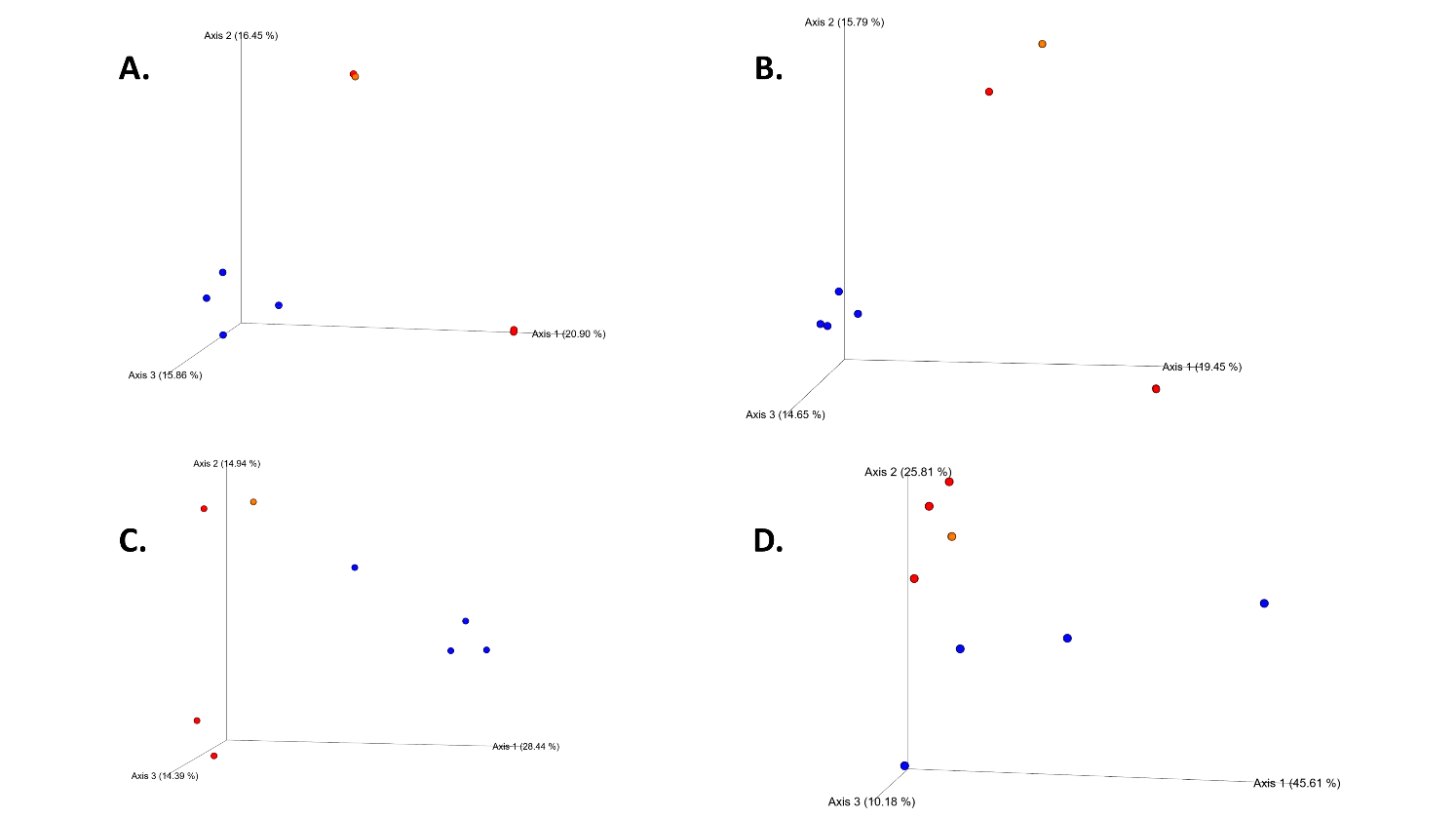


**Supplementary Figure 7. :** Sediment beta-diversity pH plots of water in the geothermal sites: A. Bray-Curtis, B. Jaccard, C. Unweighted UniFrac, D. Weighted UniFrac. Red spheres indicate high pH geothermal sites: Unnamed Hot Spring 1, Palette, Mushroom and Chocolate Pots hot springs and blue spheres indicate the low pH geothermal sites: Black Dragons Caldron, Sour Lake, Green Dragon, and Emerald Springs


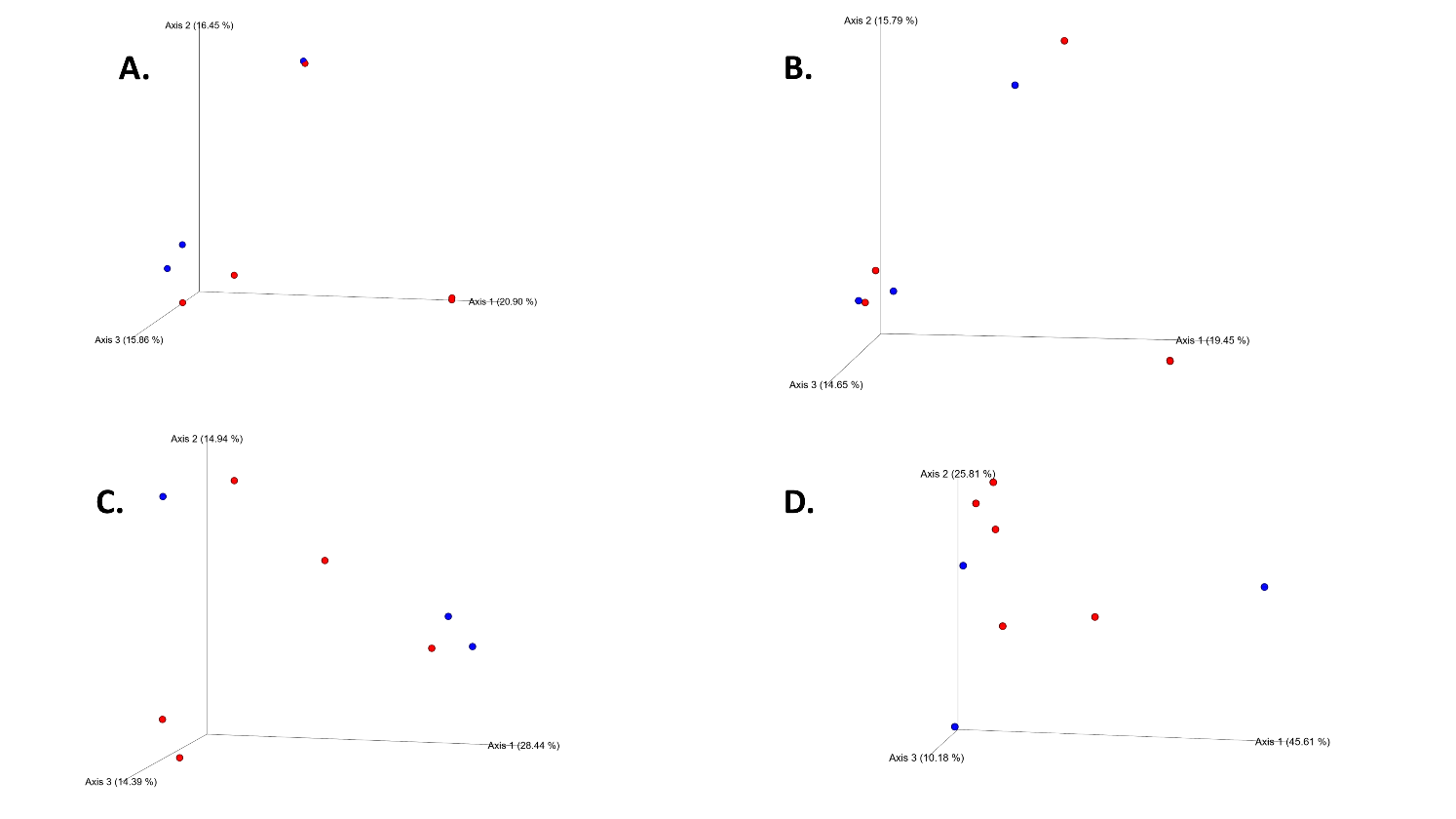


**Supplementary Figure 8. :** Sediment beta-diversity temperature plots of water in the geothermal sites: A. Bray-Curtis, B. Jaccard, C. Unweighted UniFrac, D. Weighted UniFrac. Red spheres indicate high temperature geothermal sites: Green Dragon, Unnamed Hot Spring 1, Emerald, Mushroom and Chocolate Pots hot springs and blue spheres indicate the low temperature geothermal sites: Black Dragon’s Caldron, Palette Hot Springs, and Sour Lake.


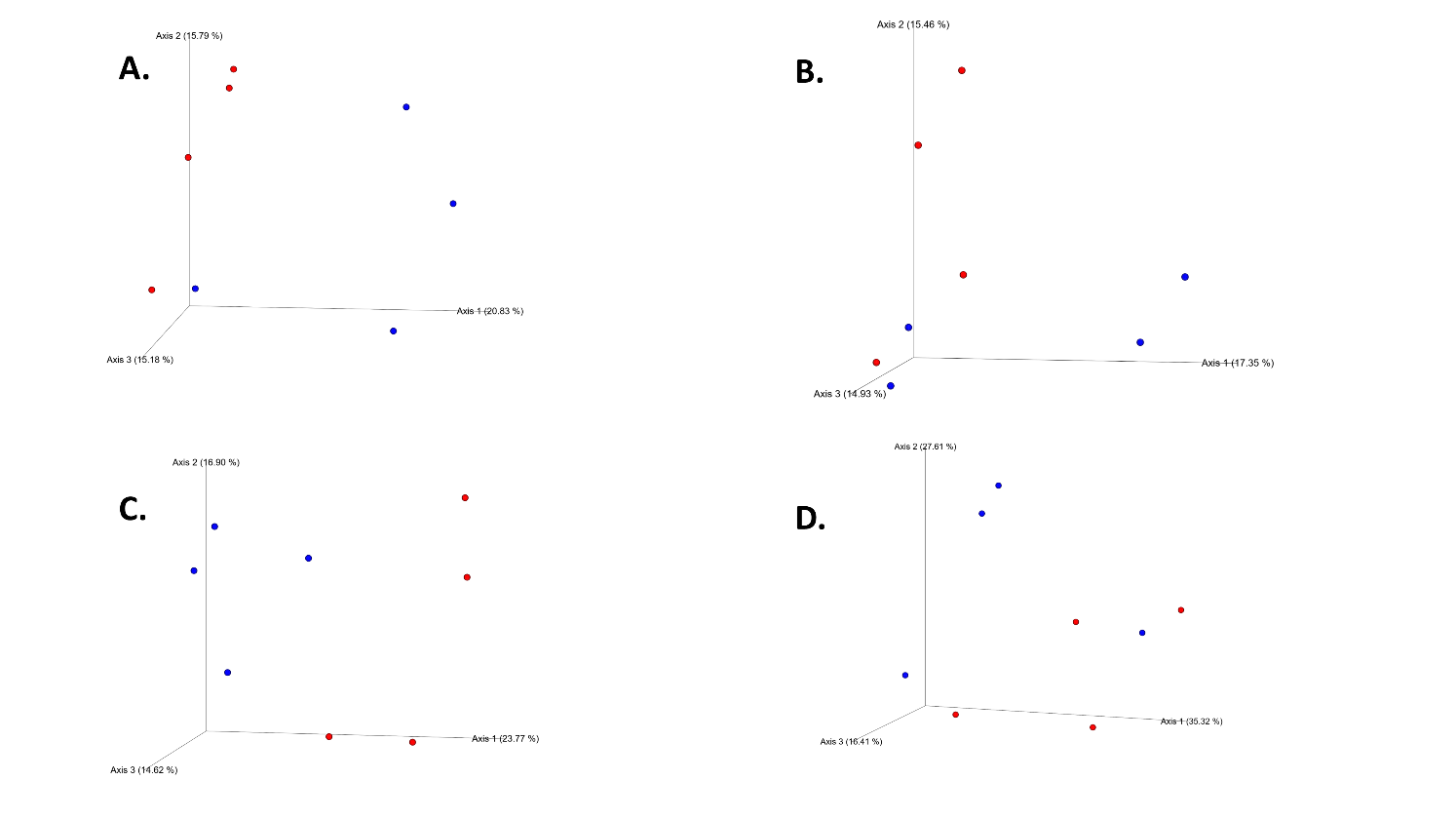


**Supplementary Figure 9.** Water beta-diversity pH plots of water in the geothermal sites: A. Bray-Curtis, B. Jaccard, C. Unweighted UniFrac, D. Weighted UniFrac. Red spheres indicate high pH geothermal sites: Unnamed Hot Spring 1, Palette, Mushroom and Chocolate Pots hot springs and blue spheres indicate the low pH geothermal sites: Black Dragons Caldron, Sour Lake, Green Dragon, and Emerald Springs.


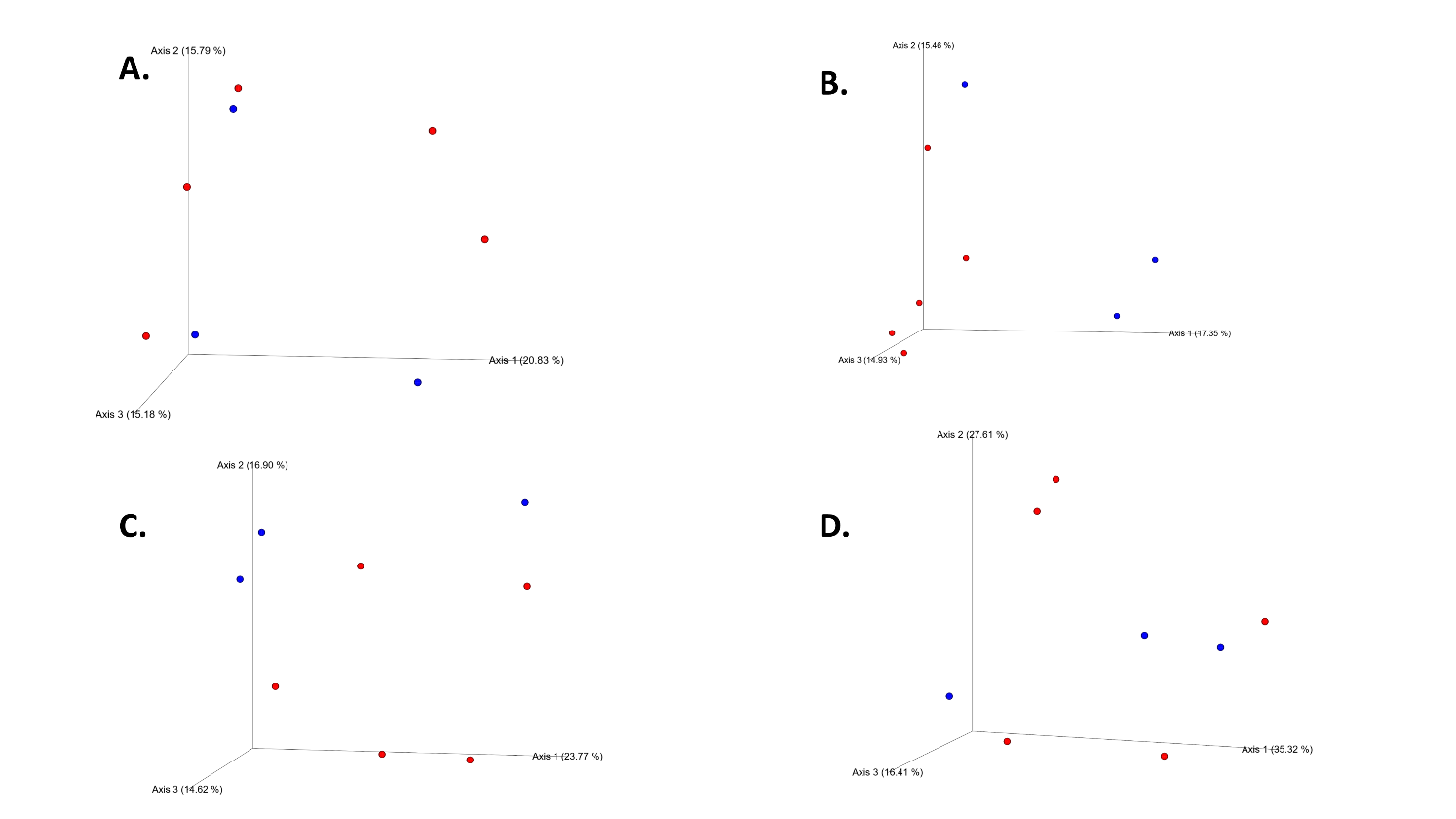


**Supplementary Figure 10.** Water beta-diversity temperature plots of water in the geothermal sites: A. Bray-Curtis, B. Jaccard, C. Unweighted UniFrac, D. Weighted UniFrac. Red spheres indicate high temperature geothermal sites: Green Dragon, Unnamed Hot Spring 1, Emerald, Mushroom and Chocolate Pots hot springs and blue spheres indicate the low temperature geothermal sites: Black Dragon’s Caldron, Palette Hot Springs, and Sour Lake.
